# NiCo_2_O_4_/FeMoO_4_ heterostructure as a bifunctional electrocatalyst for hydrogen and oxygen evolution reactions

**DOI:** 10.1039/d6ra06110a

**Published:** 2026-08-19

**Authors:** Muhammad Tahir, Mohsin Javed, Suniya Shahzad, Shahid Iqbal, Mustafa Tuzen, Afzal Shah

**Affiliations:** a Department of Chemistry, Quaid-i-Azam University Islamabad 45320 Pakistan afzals_qau@yahoo.com; b Department of Chemical and Environmental Engineering, University of Nottingham Ningbo China Ningbo 315100 China; c Tokat Gaziosmanpasa University, Faculty of Science and Arts, Chemistry Department 60250 Tokat Turkey

## Abstract

Sustainable hydrogen production requires low-cost, efficient electrocatalysts to replace precious-metal benchmarks. Here, we report a heterostructured NiCo_2_O_4_/FeMoO_4_ nanocomposite that exhibits bifunctional electrocatalytic activity for the hydrogen evolution reaction (HER) and oxygen evolution reaction (OER) in a three-electrode half-cell configuration. Fourier-transform infrared (FTIR) spectroscopy, X-ray diffraction (XRD), scanning electron microscopy (SEM), energy-dispersive X-ray spectroscopy (EDS), and X-ray photoelectron spectroscopy (XPS) analyses confirmed heterostructure formation between conductive NiCo_2_O_4_ and redox-active FeMoO_4_ bearing multivalent metal species. Among the investigated NiCo_2_O_4_/FeMoO_4_ compositions, the 3 : 2 composite exhibited the highest electrochemical activity, demonstrating that the relative composition of the two components strongly influences the HER and OER response, requiring an overpotential of only 40 mV at 10 mA cm^−2^ for the HER, with a Tafel slope of 35.9 mV dec^−1^ and a charge-transfer resistance of 2.251 Ω, as determined by electrochemical impedance spectroscopy (EIS). The OER exhibited an overpotential of 330 mV at 10 mA cm^−2^, a Tafel slope of 63 mV dec^−1^ and a charge-transfer resistance of 19.31 Ω. The electrochemically active surface area (ECSA) indicates a higher number of accessible catalytic sites. The results demonstrate the NiCo_2_O_4_/FeMoO_4_ heterostructure as a low-cost and bifunctional electrocatalyst for HER/OER and establish heterostructure engineering as an effective strategy for the development of non-precious-metal catalysts for prospective water splitting technologies.

## Abstract 2

Sustainable hydrogen production requires low-cost, efficient electrocatalysts to replace precious-metal benchmarks.

## Graphical abstract

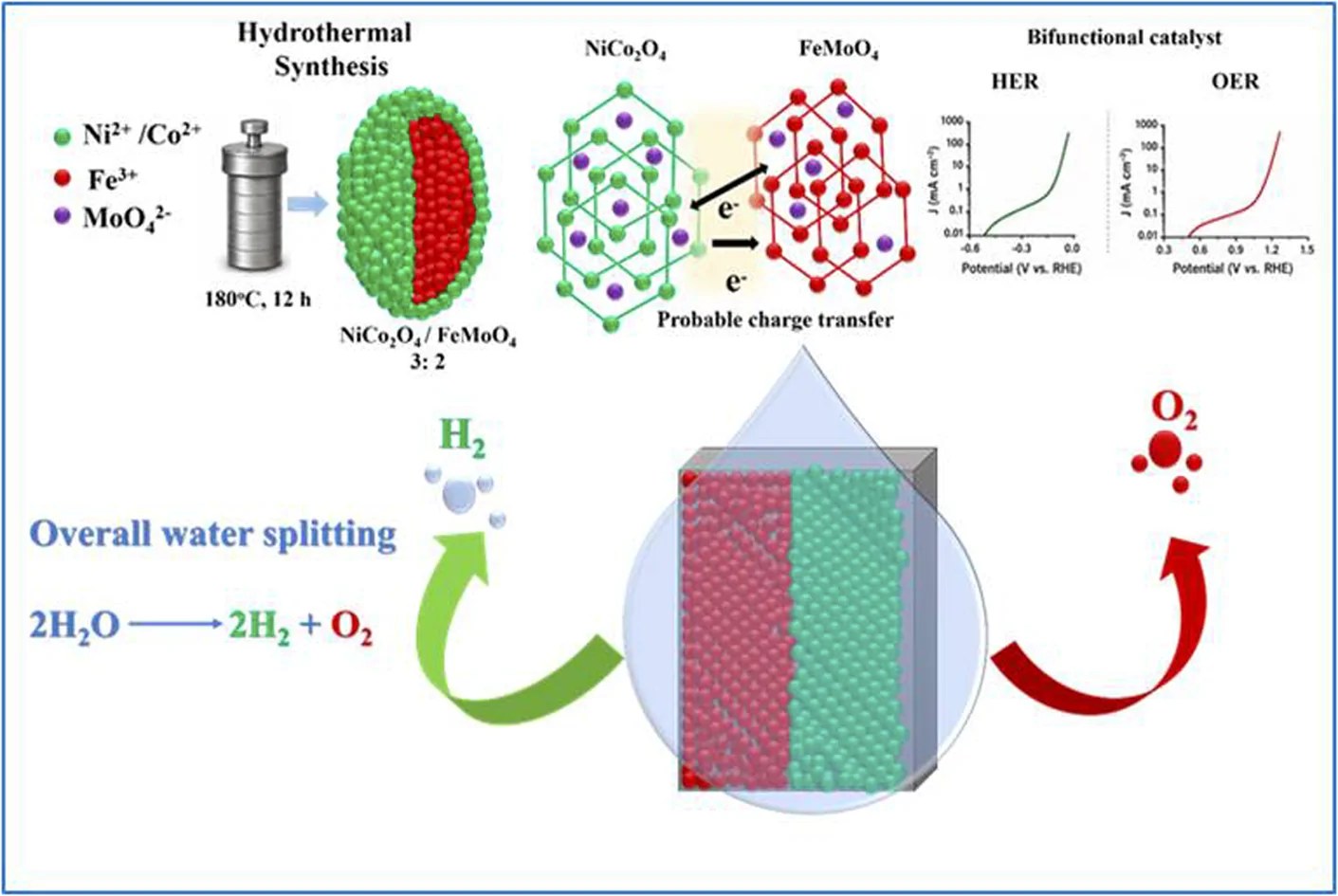

## 1. Introduction

Electrochemical water splitting has emerged as a promising approach for sustainable hydrogen production.^1^ However, the efficiency of operation is mainly restricted by the sluggish kinetics of the oxygen evolution reaction (OER) and, to a lesser extent, the hydrogen evolution reaction (HER).^2^ Benchmark noble-metal catalysts (Pt, RuO_2_, IrO_2_) are the most active for these reactions, but their scarcity and exorbitant cost hinder their widespread use, motivating continued efforts toward the development of efficient, abundant alternatives. Spinel NiCo_2_O_4_ is among the most widely studied non-noble candidates, with its mixed Ni^2+^/Ni^3+^ and Co^2+^/Co^3+^ valence states imparting comparatively high electrical conductivity.^3^ However, it typically suffers from limited exposure of active sites, and its overpotentials remain well above noble-metal benchmarks even after defect engineering, doping, or morphology control.^4^ Iron molybdate (FeMoO_4_) offers a complementary profile: its Fe^2+^/Fe^3+^ redox couple and Mo-centered coordination environment provide abundant redox-active sites, though its poor intrinsic electrical conductivity results in high charge-transfer resistance and large Tafel slopes.^5^ The limitations of the two materials are thus complementary. NiCo_2_O_4_ offers conductivity but not sufficient active-site accessibility. FeMoO_4_ has redox-active sites but not enough conductivity. Therefore, the combination of the two is a rational approach to overcoming both drawbacks simultaneously.^6^

We have an increasingly large body of modern studies that support this. On the molybdate side, Iyyappan and Ghosh engineered a Fe_2_(MoO_4_)_3_–MoO_3_ heterostructure that reduced the OER overpotential to 252–282 mV and the HER overpotential to 83–236 mV (10–100 mA cm^−2^), with stabilities exceeding 85 h and 45 h, respectively, far surpassing pristine MoO_3_.^7^ Pradhan *et al.* similarly doped Mn into a FeMoO_4_/NiMoO_4_ nanocomposite (Mn-FNMO), achieving a 284 mV OER overpotential and 107/198 mV HER overpotentials, both well below those of the parent phases.^8^ In both cases, the improved performance was attributed to interfacial electronic interaction inferred from XPS analysis; however, the conductive hosts paired with FeMoO_4_ in these studies (MoO_3_ or NiMoO_4_) are only moderately conductive compared with spinel NiCo_2_O_4_.^9^ On the NiCo_2_O_4_ side, Kong *et al.* showed that electrodepositing a FeCoNi layered double hydroxide onto NiCo_2_O_4_ nanoflowers reduced the OER overpotential from 292 mV (bare NiCo_2_O_4_) to 164 mV, with a physical mixture of the same phases failing to reproduce this gain, consistent with a genuine heterointerface effect rather than a simple additive one.^10^ Ramasamy *et al.* interfaced WO_3_, NiCo_2_O_4_, and multiwalled carbon nanotubes, lowering the OER overpotential from 501/408 mV (WO_3_/NiCo_2_O_4_ alone) to 323 mV.^11^ Even in a supercapacitor context, Zhou *et al.* showed that growing MoSe_2_ on NiCo_2_O_4_ establishes a Mo → Ni → Co electron-transfer chain that more than sextuples the specific capacitance.^12^ Collectively, these studies indicate that heterostructure engineering of NiCo_2_O_4_ with complementary functional materials can enhance electrocatalytic performance.^13^ However, direct integration of NiCo_2_O_4_ with FeMoO_4_ as a bifunctional HER/OER heterostructure remains largely unexplored. In most related reports, interfacial effects are inferred indirectly from techniques such as XRD, SEM-EDS, and XPS,^14^ while bifunctional HER/OER activity is typically reported from separate half-cell studies conducted under different conditions rather than within a single, consistent experimental protocol.

To the best of our knowledge, this is among the first studies to directly pair the high intrinsic conductivity of spinel NiCo_2_O_4_ with the redox-rich FeMoO_4_ phase within a single, hydrothermally grown heterostructure.^15^ A systematic NiCo_2_O_4_ : FeMoO_4_ mass-ratio screening (3 : 1, 3 : 2, 3 : 3) was carried out to identify the composition that best balances conductivity against active-site density, rather than adopting an arbitrary ratio. The same interface was benchmarked for both HER and OER within a single study, using a common electrode platform and characterization protocol, rather than inferring bifunctionality from separate literature reports. The optimized catalyst achieves an HER overpotential of just 40 mV at 10 mA cm^−2^ (Tafel slope: 35.9 mV dec^−1^), lower than Fe_2_(MoO_4_)_3_–MoO_3_ (83 mV) or Mn-FNMO (107 mV), while its OER performance (330 mV, 63 mV dec^−1^) is comparable though not uniformly superior to more elaborate ternary or doped heterostructures.^7,8^ This indicates that a simple, undoped two-component interface can deliver competitive performance despite its simpler binary composition, comparable to several recently reported NiCo_2_O_4_- and FeMoO_4_-based electrocatalysts and suggests a potential benefit of combined effect of the two phases directly rather than relying solely on doping or additional conductive additives.^16^ Building on this rationale, the present study synthesizes a NiCo_2_O_4_/FeMoO_4_ composite *via* a hydrothermal route, systematically varies the NiCo_2_O_4_ : FeMoO_4_ mass ratio, and characterizes the resulting materials by XRD, FTIR, SEM-EDS, and XPS. HER and OER performance is then evaluated separately in acidic and alkaline half-cell measurements, respectively, to determine whether integrating NiCo_2_O_4_ and FeMoO_4_ can address the individual limitations of each component and enhance HER/OER activity relative to the parent materials.

## 2. Experimental section

### 2.1 Materials

Fe(NO_3_)_2_·9H_2_O, Ni(NO_3_)_2_·6H_2_O, Co(NO_3_)_2_·6H_2_O, (NH_4_)_6_Mo_7_O_24_·4H_2_O, urea, potassium hydroxide, nitric acid, ethylene glycol, ethanol, methanol, acetone, and dimethylformamide (DMF) were purchased from Sigma-Aldrich and used without further purification.

### 2.2 Synthesis of NiCo_2_O_4_

NiCo_2_O_4_ was prepared by employing a urea-assisted hydrothermal process. A homogeneous solution was prepared by dissolving Co(NO_3_)_2_·6H_2_O and Ni(NO_3_)_2_·6H_2_O in deionized water (35 mL), which was then mixed with urea using continuous magnetic stirring. Following 1 hour of mixing, the solution was placed into autoclave and heated to 130 °C for 12 hours. Upon cooling to ambient temperature, the precipitate underwent centrifugation, followed by several times washing with deionized water and ethanol, and then dried at 60 °C.^17^ The resulting precursor was further calcined at 400 °C for 2 h at 3 °C min^−1^ to yield crystalline NiCo_2_O_4_. A series of nickel-cobalt oxide catalysts with varying Ni : Co molar ratios (0 : 1, 1 : 0, 1 : 1, 1 : 2, 2 : 1, 3 : 1, and 1 : 3) was synthesized at constant total metal concentration to examine the composition effect on electrocatalytic performance. Among the prepared catalysts, the Ni : Co (1 : 2) ratio exhibited the highest electrocatalytic activity and was therefore selected for further composite fabrication.

### 2.3 Synthesis of FeMoO_4_

FeMoO_4_ was synthesized by a solvothermal method. Fe(NO_3_)_2_·3H_2_O (3.5 mmol) was dissolved in ethylene glycol, and 2 mL nitric acid was added to improve precursor solubility. In a separate container, ammonium heptamolybdate tetrahydrate was dissolved in ethylene glycol to obtain a clear solution. The two solutions were combined and stirred for 30 min to form a homogeneous mixture, transferred to an autoclave, and heated at 130 °C for 12 h.^18^ After natural cooling to room temperature, the product was collected by centrifugation, washed several times with ethanol and deionized water, and dried overnight at 80 °C to obtain FeMoO_4_ powder.

### 2.4 Synthesis of NiCo_2_O_4_/FeMoO_4_

NiCo_2_O_4_/FeMoO_4_ heterostructured composites were prepared by a secondary hydrothermal growth process. Iron nitrate and ammonium molybdate were dissolved in deionized water under magnetic stirring at a Fe : Mo molar ratio of 1 : 1. The pre-synthesized NiCo_2_O_4_ powder was added to the precursor solution to form a homogeneous suspension by ultrasonication and stirring.^17^ The mass ratio of NiCo_2_O_4_ to FeMoO_4_ (3 : 1, 3 : 2, or 3 : 3) was controlled by adjusting the precursor concentrations. The suspension was transferred to an autoclave for hydrothermal treatment to grow FeMoO_4_*in situ* on the NiCo_2_O_4_ surface. After the reaction, the product was collected by centrifugation, rinsed several times, and dried. The resulting composite was then annealed to enhance crystallinity and strengthen the heterostructured interface.

### 2.5 Working electrode preparation

The catalyst ink was prepared by dispersing 5 mg of catalyst in 300 µL of dimethylformamide (DMF) under ultrasonication to obtain a homogeneous suspension without the addition of a polymeric binder. An aliquot of 15 µL of the ink, corresponding to a catalyst loading of 0.25 mg (≈3.54 mg cm^−2^), was drop-cast onto the polished surface of a glassy carbon electrode (GCE, 3 mm diameter, geometric area 0.0707 cm^2^) and dried naturally at room temperature.^19^ The high boiling point of DMF allowed slow, uniform solvent evaporation, yielding a compact, well-adhered catalyst film without the need for a binder.^20^ The same procedure and loading were applied to all catalysts (NiCo_2_O_4_, FeMoO_4_, and the NiCo_2_O_4_/FeMoO_4_ composites) to ensure a fair and direct comparison of their electrocatalytic activities; a systematic investigation of the effect of catalyst loading was beyond the scope of the present study.

The glassy carbon electrode was modified with a catalyst loading of 0.25 mg, which was kept constant for all electrochemical measurements. This loading was selected as a practical working condition to obtain a reproducible and sufficiently uniform catalyst layer while avoiding excessive film thickness that could adversely affect charge transfer and mass transpor.^21^ The same catalyst loading was used for all investigated samples to ensure a consistent basis for comparison. Therefore, 0.25 mg is regarded as the standardized working loading rather than an experimentally optimized value.

### 2.6 Characterization

X-ray diffraction (XRD) patterns were recorded using a PANalytical X'Pert PRO diffractometer (model 3040/60).^22^ Compositional analysis was performed using a Nicolet Summit Fourier-transform infrared (FTIR) spectrometer, and the materials were further characterized by field-emission scanning electron microscopy (SEM), energy-dispersive X-ray spectroscopy (EDS), and X-ray photoelectron spectroscopy (XPS).

Electrochemical measurements were performed using a Gamry Interface 5000E workstation in a three-electrode configuration, with a GCE (3 mm diameter) as the working electrode, a platinum wire as the counter electrode, and Ag/AgCl as the reference electrode.^23^ HER was evaluated in 0.5 M H_2_SO_4_ and OER in 1.0 M KOH, with linear sweep voltammetry (LSV) recorded at a scan rate of 10 mV s^−1^.^24^ EIS measurements were performed over a frequency range of 100 kHz to 0.1 Hz using a sinusoidal AC perturbation amplitude of 10 mV rms.^25^ The spectra were recorded at a DC potential of −0.50 V *vs.* Ag/AgCl for HER and +1.50 V *vs.* Ag/AgCl for OER and fitted to an equivalent-circuit model (*R*_s_ − (*R*_ct_‖CPE) − *W*_d_, where *R*_s_ is the solution resistance, *R*_ct_ the charge-transfer resistance, CPE the constant phase element, and *W*_d_ the Warburg diffusion element; Tables S3 and S4) using Gamry Echem Analyst.^26^ For graphical presentation and electrochemical comparison, potentials were converted from the Ag/AgCl reference scale used during measurement to the reversible hydrogen electrode (RHE) scale using the following equation:*E*_RHE_ = *E*_Ag/AgCl_ + 0.197 + 0.059 × pH

Electrochemical stability was assessed by chronoamperometry at fixed applied potentials of +1.50 V *vs.* Ag/AgCl for OER (1 M KOH) and −0.50 V *vs.* Ag/AgCl for HER (0.5 M H_2_SO_4_), with the current density monitored continuously for approximately 24 h in each case.^27^ LSV curves were recorded before and after each stability test at a scan rate of 10 mV s^−1^ to evaluate any change in the polarization response.^28^

The double-layer capacitance (*C*_dl_) was evaluated from the non-faradaic region of the cyclic voltammograms (CV),^29^ using the slope of capacitive current density (Δ*j* = *j*_a_ − *j*_c_)/2 plotted against scan rate (*ν*), according to:
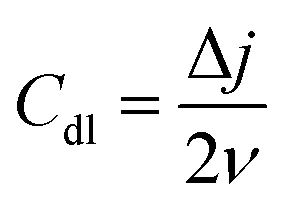


The electrochemically active surface area (ECSA) was assessed from the relation:
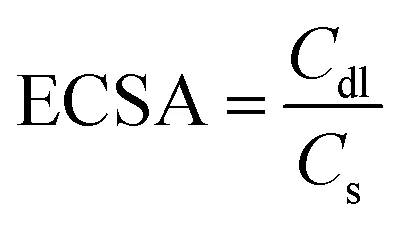
where *C*_s_ is the specific capacitance of a flat surface of uniform composition under the same electrolyte conditions.^30^ A value of 0.040 mF cm^−2^ was used for *C*_s_, as is conventional for electrocatalysts tested in alkaline media (1.0 M KOH).^31^ The resulting ECSA values therefore represent a relative measure of the electrochemically accessible surface area of the catalysts, rather than their absolute total surface area.

The roughness factor (RF), which reflects the surface area of the catalyst relative to a smooth electrode, was calculated as:
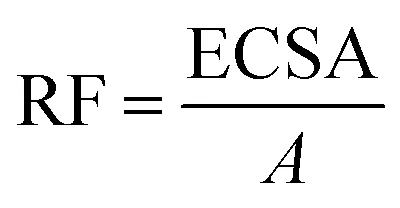
where *A* stands for the electrode's geometric surface area.^32^

The intrinsic catalytic activity was assessed by calculating the turnover frequency (TOF) for the OER and HER using:
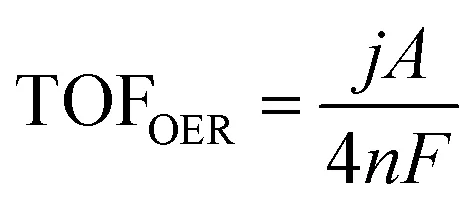

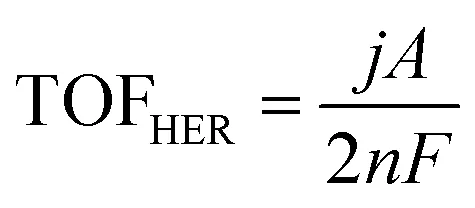
where *j* is the current density, *A* is the electrode's geometric surface area (used to convert current density into total current), *n* is the number of moles of catalytically active sites, and *F* is the Faraday constant; the factors of 4 and 2 correspond to the number of electrons transferred in the OER and HER mechanisms, respectively.^33^ The number of active sites was estimated by assuming a surface active-site density of 1 × 10^−9^ mol cm^−2^ (Section 3.2), since the exact number of electrochemically active sites is difficult to determine experimentally and may not scale directly with total metal content; all electrochemically accessible metal atoms were assumed to act as equally active catalytic sites, a common approximation in comparative studies. Therefore, the obtained TOF should be regarded as an estimated or apparent value rather than an absolute intrinsic activity. The lower TOF of the 3 : 2 NiCo_2_O_4_/FeMoO_4_ composite compared to pristine FeMoO_4_ could be related to the larger estimated population of electrochemically active sites in the composite, which distributes the measured catalytic current over more estimated sites. Therefore, the TOF values should be addressed in conjunction with the total HER activity and ECSA rather than a direct indicator of the total catalytic performance.^34^ The number of active sites, *n*, was in turn obtained as *n* = *Γ* × ECSA, where *Γ* is the assumed surface-active-site density given above and ECSA is the electrochemically active surface area (Section 2.6), rather than from the geometric electrode area *A*. The ECSA values used for the OER TOF calculation were those obtained from *C*_dl_ measurements in 1.0 M KOH (Section 3.3), consistent with the alkaline medium in which OER was measured. Because HER was measured in 0.5 M H_2_SO_4_ while the reported ECSA/*C*_dl_ data are associated with the alkaline (1.0 M KOH) measurements, and no separate acid-medium ECSA dataset is reported in this study, the basis for applying this ECSA value to the HER TOF calculation cannot be confirmed from the data presented and is noted here as requiring author verification. The reported TOF values should therefore be regarded as apparent, rather than absolute, kinetic parameters; relative comparisons between catalysts nonetheless remain valid, since identical assumptions and calculation procedures were applied to all samples.

The mass activity (MA) was calculated to assess catalyst utilization efficiency according to:
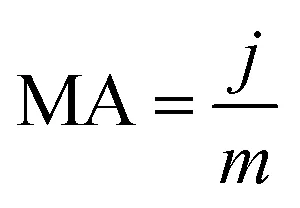
where *m* is the catalyst mass loaded on the electrode surface.^35^

## 3. Results and discussion

### 3.1 Structural characterizations

The rationale for combining NiCo_2_O_4_ and FeMoO_4_ into a single electrode architecture is that the two phases are expected to complement one another: the spinel NiCo_2_O_4_ component is well established as an active, conductive oxide framework, while the FeMoO_4_ phase is expected to contribute additional redox-active centers and, being a comparatively robust oxide, additional structural stability to the composite.^5,35^ Formation of a junction between the two phases may also introduce local lattice distortion at the phase boundaries; whether this is accompanied by oxygen-vacancy formation or gives rise to additional catalytically relevant sites cannot be established from the present dataset and would require direct nanoscale probes (*e.g.*, TEM/HRTEM) beyond the characterization performed here. The extent to which this design rationale is realized is examined below, first through the structural and chemical characterization of the composite, and subsequently through its electrochemical performance (Sections 3.2 and 3.3).

**Fig. 1:**
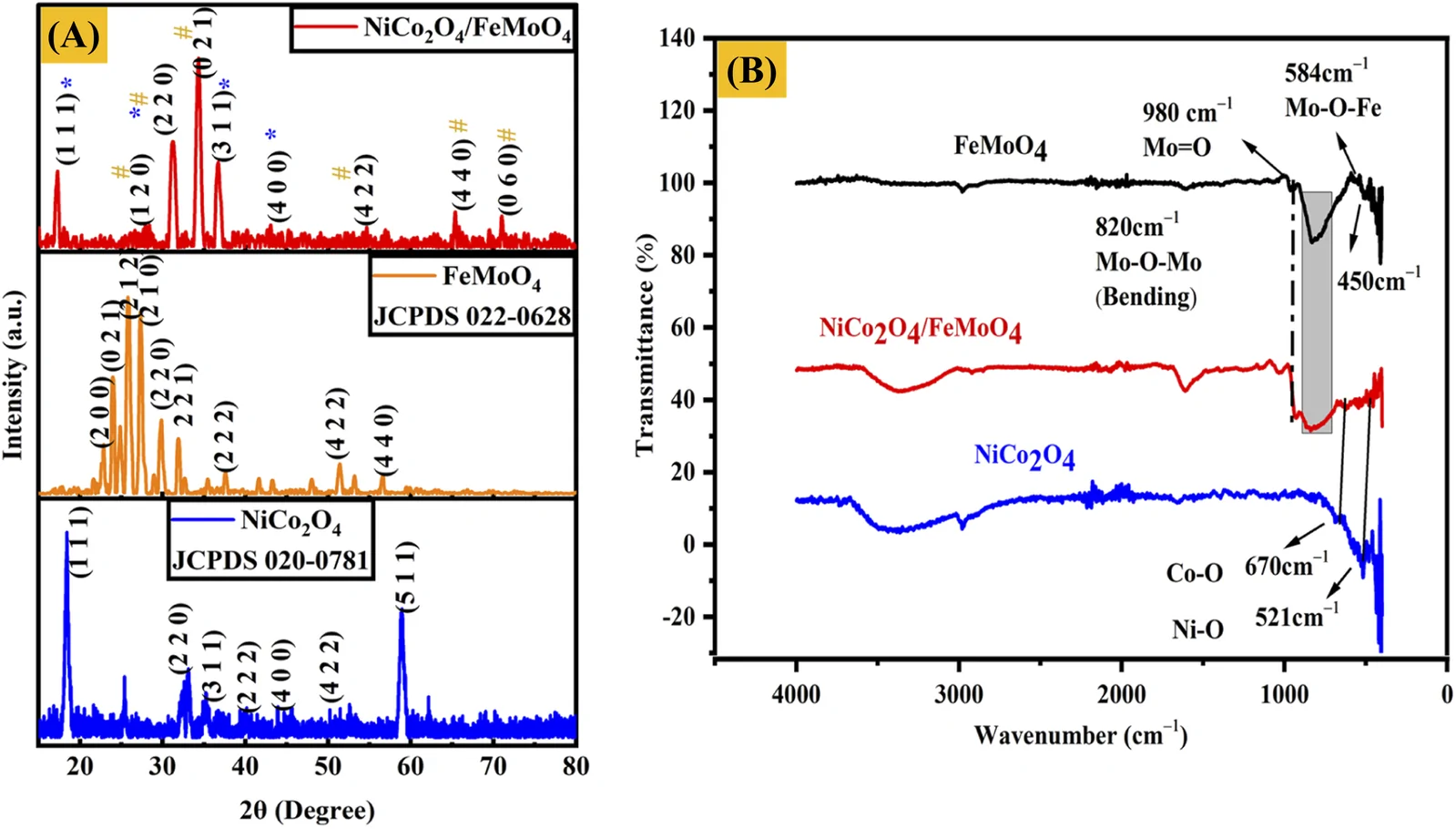
(A) XRD patterns and (B) FTIR spectra of NiCo_2_O_4_, FeMoO_4_, and NiCo_2_O_4_/FeMoO_4_ composite.

XRD was used to assess the crystallinity and phase purity of the catalytic materials, with the patterns of pure FeMoO_4_, pure NiCo_2_O_4_, and the NiCo_2_O_4_/FeMoO_4_ composite compared in Fig. 1(A). The diffraction peaks of FeMoO_4_ (middle panel) match well with the standard pattern for monoclinic FeMoO_4_ (JCPDS card no. 022-0628), with prominent reflections indexed to the (200), (021), (212), (210), (220), (221), (222), (422), and (440) planes and no detectable secondary impurity phases, consistent with successful formation of phase-pure FeMoO_4_.^36^ The pure NiCo_2_O_4_ pattern (lower panel) likewise agrees with the reference pattern for cubic spinel NiCo_2_O_4_ (JCPDS card no. 020-0781, *Fd*3̄*m* space group).

For pure NiCo_2_O_4_, the major diffraction peaks located at 2*θ* ≈ 18.7°, 31.5°, 36.6°, 43.6°, and 59.3° are indexed to the (111), (220), (311), (400), and (511) planes, respectively, with additional reflections corresponding to the (222) and (422) planes also observed, consistent with the well-crystallized nature of the NiCo_2_O_4_ phase.^37^ In the NiCo_2_O_4_/FeMoO_4_ composite (top panel), the characteristic reflections of both NiCo_2_O_4_ and FeMoO_4_ are retained side by side, with no additional peaks that would point to a secondary crystalline phase, consistent with coexistence of the two phases rather than formation of a new compound. This retention of the individual diffraction patterns indicates that each component preserves its own crystal structure within the composite. The modest changes in relative peak intensity and the slight peak broadening observed for the composite may be associated with micro-strain arising from lattice mismatch at the NiCo_2_O_4_–FeMoO_4_ phase boundaries; such features are consistent with structural effects such as micro-strain and local lattice distortion, but XRD data alone cannot establish electronic interaction between the two phases, and any such effects would need to be assessed by complementary techniques (*e.g.*, XPS) rather than inferred from diffraction data alone. Taken together, the phase purity, crystallinity, and peak-broadening behavior observed by XRD are consistent with successful formation of a well-defined, impurity-free NiCo_2_O_4_/FeMoO_4_ composite, providing a suitable structural basis for the electrocatalytic evaluation discussed in the following sections.

**Fig. 2:**
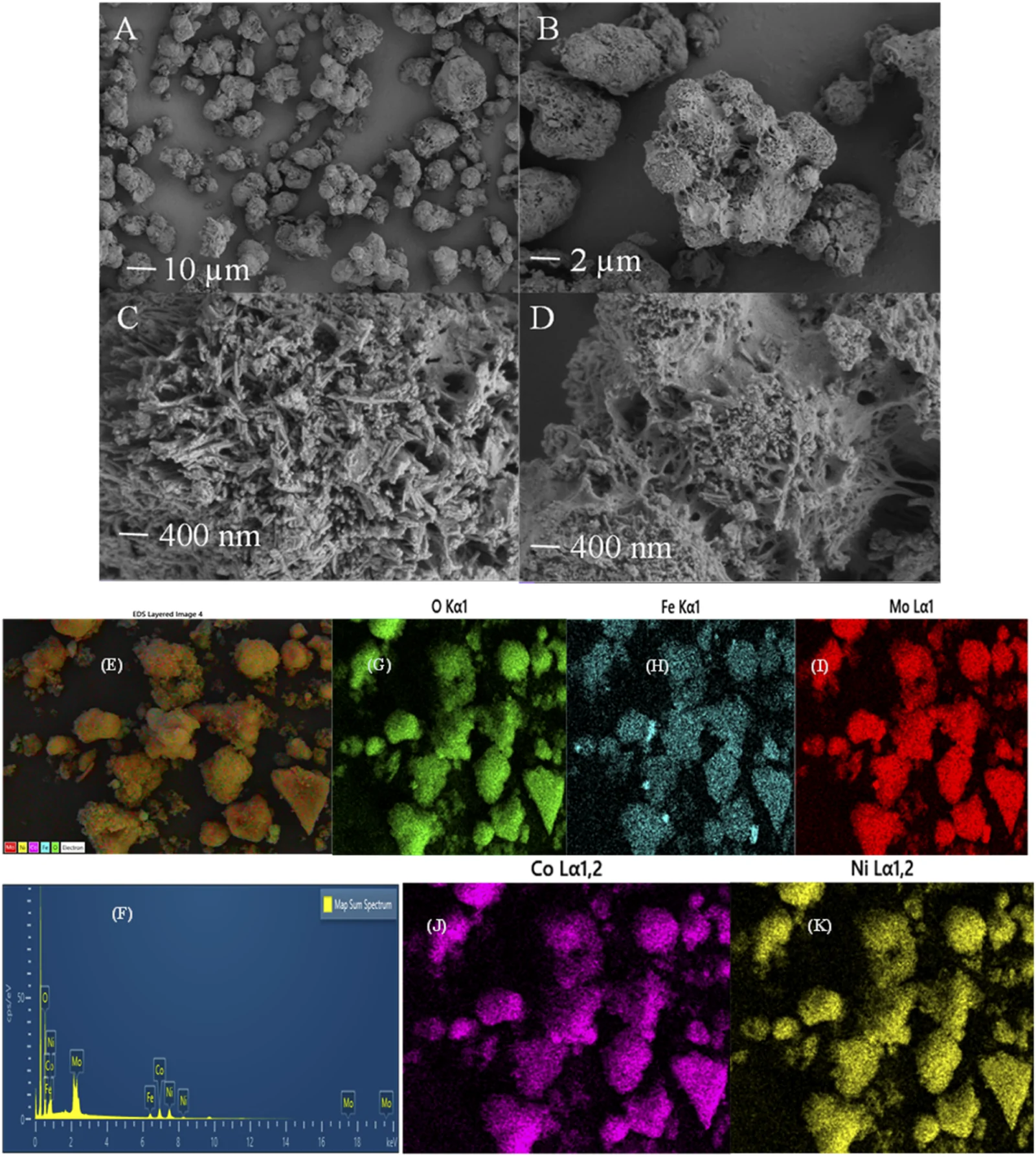
SEM images (A–D) and EDS elemental mapping of the NiCo_2_O_4_/FeMoO_4_ composite: (E) layered elemental overlay, (F) EDS sum spectrum, and the corresponding elemental maps for (G) O, (H) Fe, (I) Mo, (J) Co, and (K) Ni.

This phase coexistence identified by XRD is corroborated by FTIR (Fig. 1(B)), which probes the local vibrational environment of the metal–oxygen bonds. The spectrum of pure NiCo_2_O_4_ displays the characteristic metal–oxygen stretching vibrations expected for the spinel structure in the 400–700 cm^−1^ region, with bands at 521 and 670 cm^−1^ attributable to Ni–O and Co–O stretching, respectively.^38^ For pure FeMoO_4_, bands at 980, 820, 584, and 450 cm^−1^ are assigned to Mo=O stretching, Mo–O–Mo bending, Mo–O–Fe stretching, and Fe–O vibrational modes, respectively.^39^ The composite spectrum reproduces both sets of bands, supporting the coexistence of NiCo_2_O_4_- and FeMoO_4_-derived local bonding environments identified by XRD, without indication of new vibrational features that would suggest an additional phase. With the vibrational and diffraction signatures of both phases established, SEM was used to examine how this translates into the surface morphology of the synthesized materials. The coexistence of the characteristic crystalline phases of NiCo_2_O_4_ and FeMoO_4_, together with their uniform elemental distribution, supports the formation of the NiCo_2_O_4_/FeMoO_4_ heterostructured composite. Pure NiCo_2_O_4_ forms agglomerated particle clusters with a relatively compact morphology (Fig. S1),^40^ whereas pure FeMoO_4_ develops larger, irregular, grain-like formations with comparatively lower surface roughness (Fig. S2).^18^ The NiCo_2_O_4_/FeMoO_4_ composite, in contrast, displays a highly porous, interconnected morphology (Fig. 2(A–D)) that combines features reminiscent of both parent morphologies, consistent with intimate physical integration of the two components; identifying the elemental identity of specific features within this architecture requires the complementary EDS analysis discussed below. This porous, interconnected architecture is expected to increase the electrochemically accessible surface area and the number of exposed catalytic sites, consistent with the ECSA trends reported separately, and is generally favorable for electrolyte accessibility and ion diffusion, which may contribute to the electrocatalytic activity discussed in later sections.

**Fig. 3:**
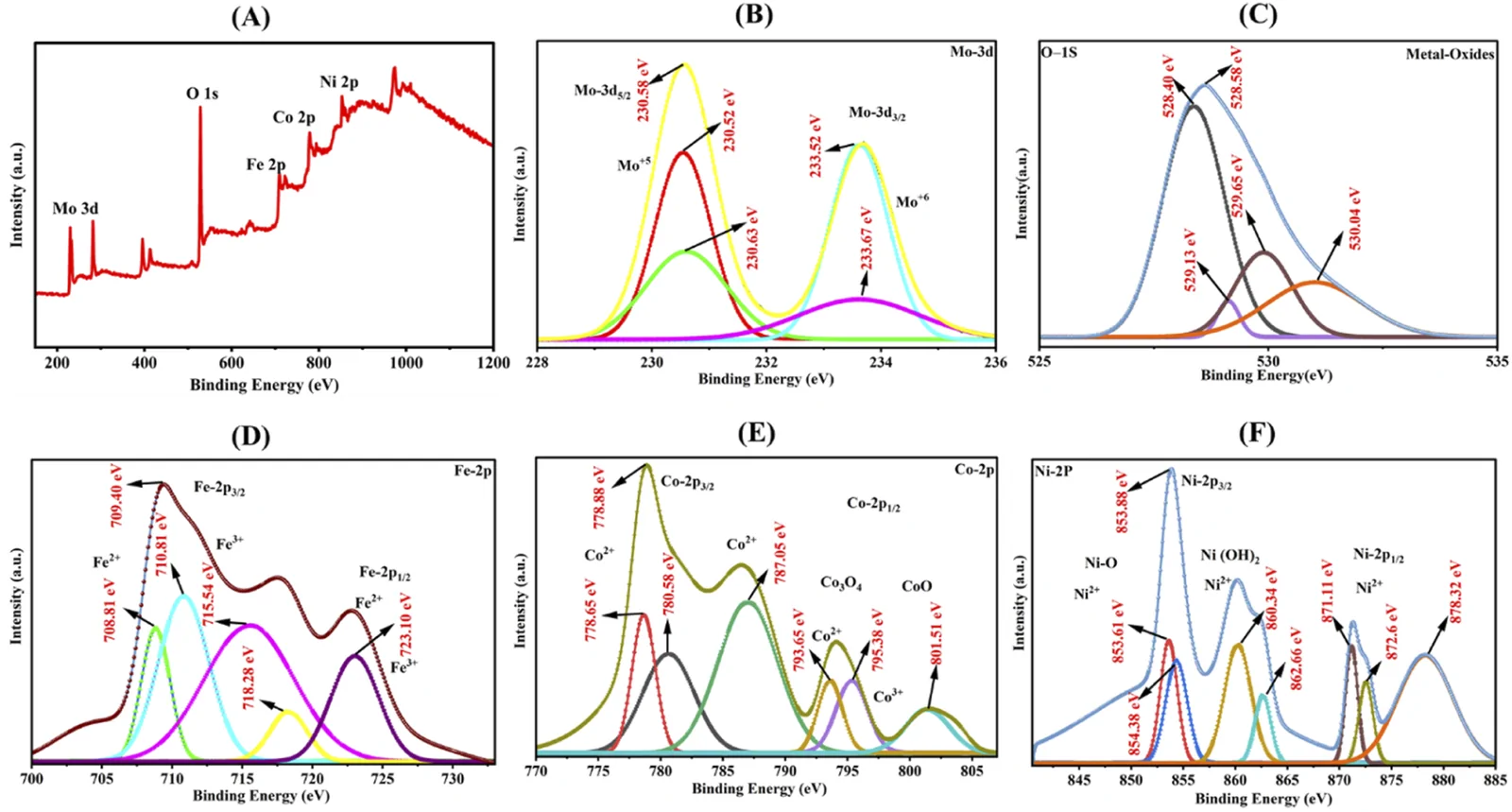
XPS spectra of (A) NiCo_2_O_4_/FeMoO_4_, along with the corresponding deconvoluted spectra of (B) Mo 3d, (C) O 1s, (D) Fe 2p, (E) Co 2p, and (F) Ni 2p.

Elemental composition was assessed by EDS to complement the morphological observations above. The EDS spectrum of pure NiCo_2_O_4_ (Fig. S3) shows the expected Ni, Co, and O signals, with weight percentages in reasonable agreement with the nominal composition,^41^ The EDS spectrum of pure FeMoO_4_ (Fig. S4) confirms the presence of Fe, Mo, and O, consistent with the phase identity established by XRD. Although some local regions in the FeMoO_4_ elemental maps exhibit weak or apparently absent signals, these features are attributed to local variations in particle topography, orientation, overlap, and X-ray collection efficiency during mapping rather than being interpreted as evidence of a distinct composition. For the composite, the EDS sum spectrum (Fig. 2(F)) shows simultaneous detection of Ni, Co, Fe, Mo, and O within the sampled region, consistent with the coexistence of NiCo_2_O_4_- and FeMoO_4_-derived phases established by XRD and FTIR. Elemental detection by EDS alone, however, cannot establish true nanoscale interfacial contact or heterostructure formation between the two phases. The quantified elemental composition of all samples shows good agreement with theoretical values. The corresponding elemental maps (Fig. 2(G–K)) show a visually uniform spatial distribution of O, Fe, Mo, Co, and Ni across the imaged region, overlaid in Fig. 2(E); as SEM-EDS mapping has a spatial resolution on the micrometer scale, this reflects the absence of large-scale elemental segregation within that resolution limit, but it does not rule out phase separation or confirm true interfacial mixing at the nanoscale, which would require higher-resolution techniques such as TEM. This relatively uniform elemental distribution, together with the porous morphology described above, is expected to improve the accessibility of catalytic sites and mass transport at the electrode surface; the resulting electrochemical behaviour, including charge-transfer characteristics, is evaluated separately by HER, OER, and EIS measurements below. Beyond bulk phase identity and elemental distribution, XPS was used to probe the surface chemical states of the composite. The survey spectrum (Fig. 3(A)) confirms the presence of Ni, Co, Fe, Mo, and O at the surface of the NiCo_2_O_4_/FeMoO_4_ composite.^6^ In the high-resolution Ni 2p spectrum (Fig. 3(F)), the deconvoluted peaks are assignable to Ni^2+^ in two distinct local environments: a Ni–O lattice-type environment (853.6–853.9 eV, 2p_3/2_) and a more hydroxylated Ni(OH)_2_-like environment (860.3–862.7 eV), together with the corresponding 2p_1/2_ features at 871.1–872.6 eV and a higher-binding-energy feature at 878.3 eV consistent with the shake-up satellite structure typical of Ni^2+^. The Co 2p spectrum (Fig. 3(E)) shows core-level signals assignable to both Co^2+^ and Co^3+^, with additional higher-binding-energy features near 787 and 801.5 eV consistent with shake-up satellites characteristic of these oxidation states. The Fe 2p spectrum (Fig. 3(D)) is dominated by Fe^3+^ signals at approximately 709.4 eV (2p_3/2_) and 723.1 eV (2p_1/2_), with smaller contributions near 708.8 eV consistent with a minor Fe^2+^ component, indicating mixed Fe valency. The Mo 3d spectrum (Fig. 3(B)) shows 3d_5/2_ and 3d_3/2_ envelopes centered at approximately 230.6 eV and 233.5 eV, respectively, each resolvable into Mo^5+^- and Mo^6+^-type components, in agreement with the presence of the FeMoO_4_ phase identified by XRD.^5^

The oxide nature of the composite is further supported by the O 1s spectrum (Fig. 3(C)), which deconvolutes into several components spanning approximately 528.4–530.0 eV. The lower-binding-energy components (approximately 528.4–529.1 eV) are attributable to lattice metal–oxygen (M–O) bonds, while the higher-binding-energy components (approximately 529.7–530.0 eV) are consistent with adsorbed oxygen species and surface hydroxyl groups. Such surface oxygen species are generally considered relevant to catalytic activity, as they are thought to assist proton adsorption and charge-transfer processes during electrocatalysis. A quantitative comparison of the binding energies of pristine NiCo_2_O_4_, pristine FeMoO_4_, and the NiCo_2_O_4_/FeMoO_4_ composite (pristine-oxide XPS spectra provided in Fig. S6 and S7) is summarized in Table S1.^42^ Relative to the pristine oxides, the composite exhibits positive binding-energy shifts of +0.27 eV for Ni 2p_3/2_, +0.47 eV for Co 2p_3/2_, +0.68 eV for Fe 2p_3/2_, +0.63 eV for Fe 2p_3/2_, and +0.51 eV for Mo 3d_3/2_. In contrast, the Ni 2p_3/2_ and Mo 3d_3/2_ peaks show only minor shifts of −0.09 and −0.06 eV, respectively. The predominantly positive shifts observed for the major core-level peaks indicate changes in the local chemical environment of the metal centers following composite formation. These shifts support interfacial chemical interaction between the NiCo_2_O_4_ and FeMoO_4_ phases. Such shifts may be associated with some degree of electronic interaction between NiCo_2_O_4_ and FeMoO_4_, but binding-energy shifts of this kind can arise from several distinct local effects, and establishing a direct, quantitative link to charge transport and electrocatalytic kinetics would require evidence beyond the binding-energy shifts alone.^43^

Collectively, the XPS results confirm the presence of Ni, Co, Fe, and Mo in oxidation states consistent with the NiCo_2_O_4_ and FeMoO_4_ phases identified by XRD, and the accompanying binding-energy shifts point to a modified local chemical environment at the phase boundaries that may contribute to the electrocatalytic behaviour examined in the following sections.

### 3.2 Catalytic performance for HER

**Fig. 4:**
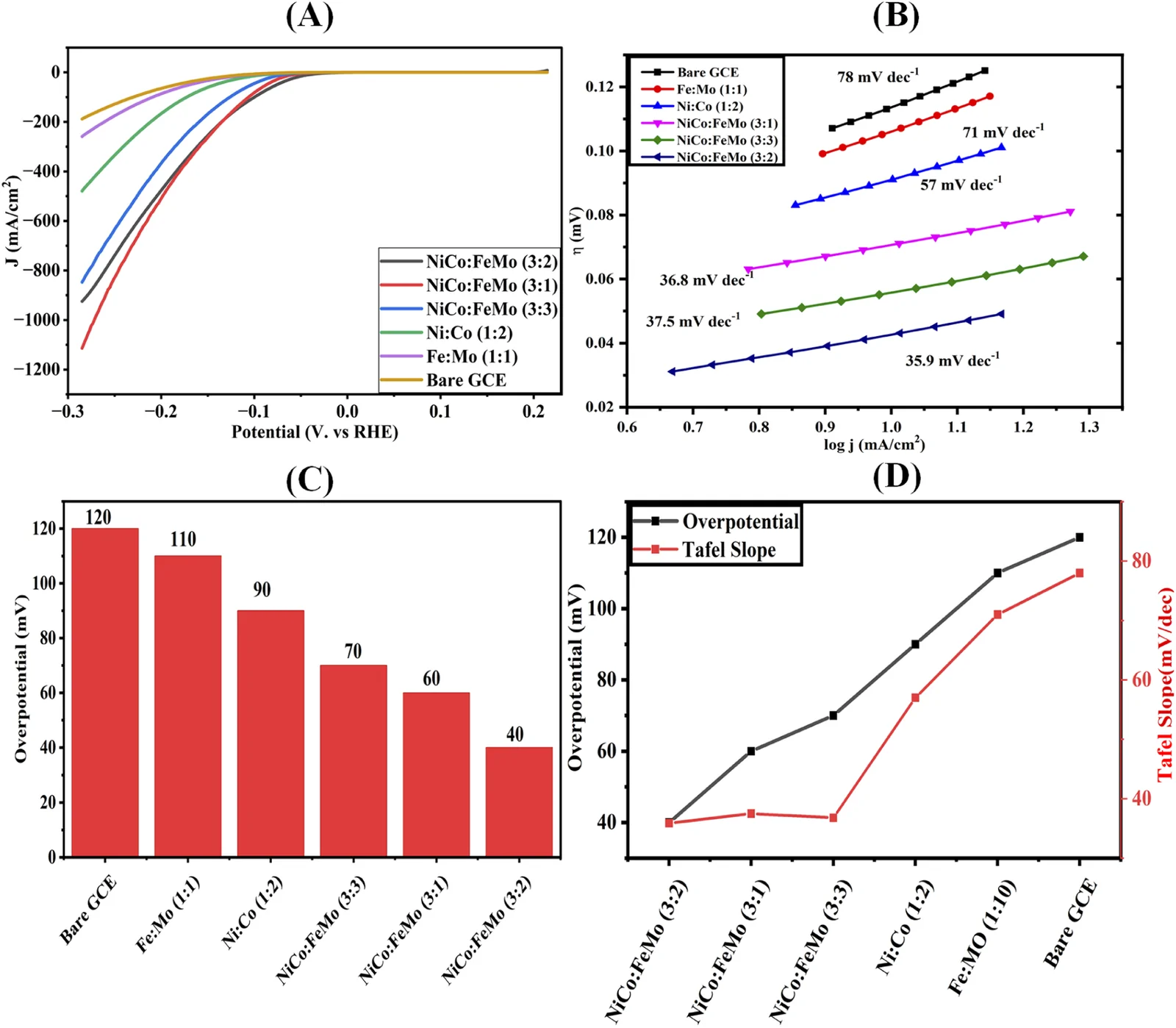
(A) LSV polarization curves, (B) Tafel kinetic analysis in 0.5 M H_2_SO_4_ at 10 mV s^−1^, and (C) comparative analysis of overpotential performance, and (D) relationship between Tafel slopes and overpotentials for bare GCE, FeMoO_4_ (1 : 1), NiCo_2_O_4_ (1 : 2), and NiCo_2_O_4_/FeMoO_4_ composites (3 : 1, 3 : 2, 3 : 3).

The HER activity of the synthesized materials was evaluated by linear scan voltammetry in 0.5 M H_2_SO_4_, comparing bare GCE, FeMoO_4_, NiCo_2_O_4_ (1 : 2), and the NiCo_2_O_4_/FeMoO_4_ composites at mass ratios of 3 : 1, 3 : 2, and 3 : 3 (Fig. 4(A)). HER activity is markedly enhanced across the NiCo_2_O_4_/FeMoO_4_ composite series relative to the individual components and bare GCE, and this enhancement is strongly composition-dependent: the 3 : 2 composite reaches 10 mA cm^−2^ at an overpotential of only 40 mV, compared with 90 mV for NiCo_2_O_4_, 110 mV for FeMoO_4_, and 120 mV for bare GCE (Fig. 4(C)), while the 3 : 1 and 3 : 3 composites require intermediate overpotentials of 60 and 70 mV, respectively (Table 1). The heterostructured catalysts also sustain higher current densities than either parent oxide, consistent with the trend in overpotential and pointing to a composition-dependent enhancement in HER activity upon heterostructure formation.

**Table 1:** EIS and HER characteristics of bare and modified GCE with Ni : Co (1 : 2), FeMoO_4_, and NiCo_2_O_4_/FeMoO_4_ composites

Electrocatalysts	*η*@10 mA cm^−2^ (mV)	*R*_s_ (Ω)	*j* (mA cm^−2^)	Tafel slope (mV dec^−1^)	Mass activity (mA mg^−1^)	*R*_ct_ (Ω)	TOF (s^−1^)
Bare GCE	120	1.858	—	78	—	5.619	—
FeMoO_4_	110	1.711	847	71	240.56	5.068	12.10
NiCo_2_O_4_	90	1.759	258.87	57	73.52	4.441	2.68
NiCo_2_O_4_/FeMoO_4_ (3 : 1)	60	1.797	1001.69	37.5	284.48	3.155	8.93
NiCo_2_O_4_/FeMoO_4_ (3 : 2)	40	1.878	479	35.9	136.04	2.251	3.91
NiCo_2_O_4_/FeMoO_4_ (3 : 3)	70	1.809	924.79	36.8	262.64	4.037	8.62

Because pristine FeMoO_4_ already shows an appreciable HER overpotential of 110 mV, FeMoO_4_ itself must be regarded as an electrochemically active component rather than a passive constituent of the composite. However, the present dataset does not allow the individual contributions of FeMoO_4_ and NiCo_2_O_4_ to be quantitatively separated, nor does it identify either phase as the sole source of active sites; NiCo_2_O_4_ may also contribute to the electrochemical response and charge-transfer behavior of the composite. The markedly lower overpotential of the 3 : 2 composite relative to either parent oxide is therefore more appropriately attributed to complementary contributions from both constituent phases at the investigated composition rather than to a single dominant component. Establishing the quantitative relationship between FeMoO_4_ content and catalytic contribution would require a systematic composition series specifically designed for this purpose, which lies beyond the scope of the present study.

**Fig. 5:**
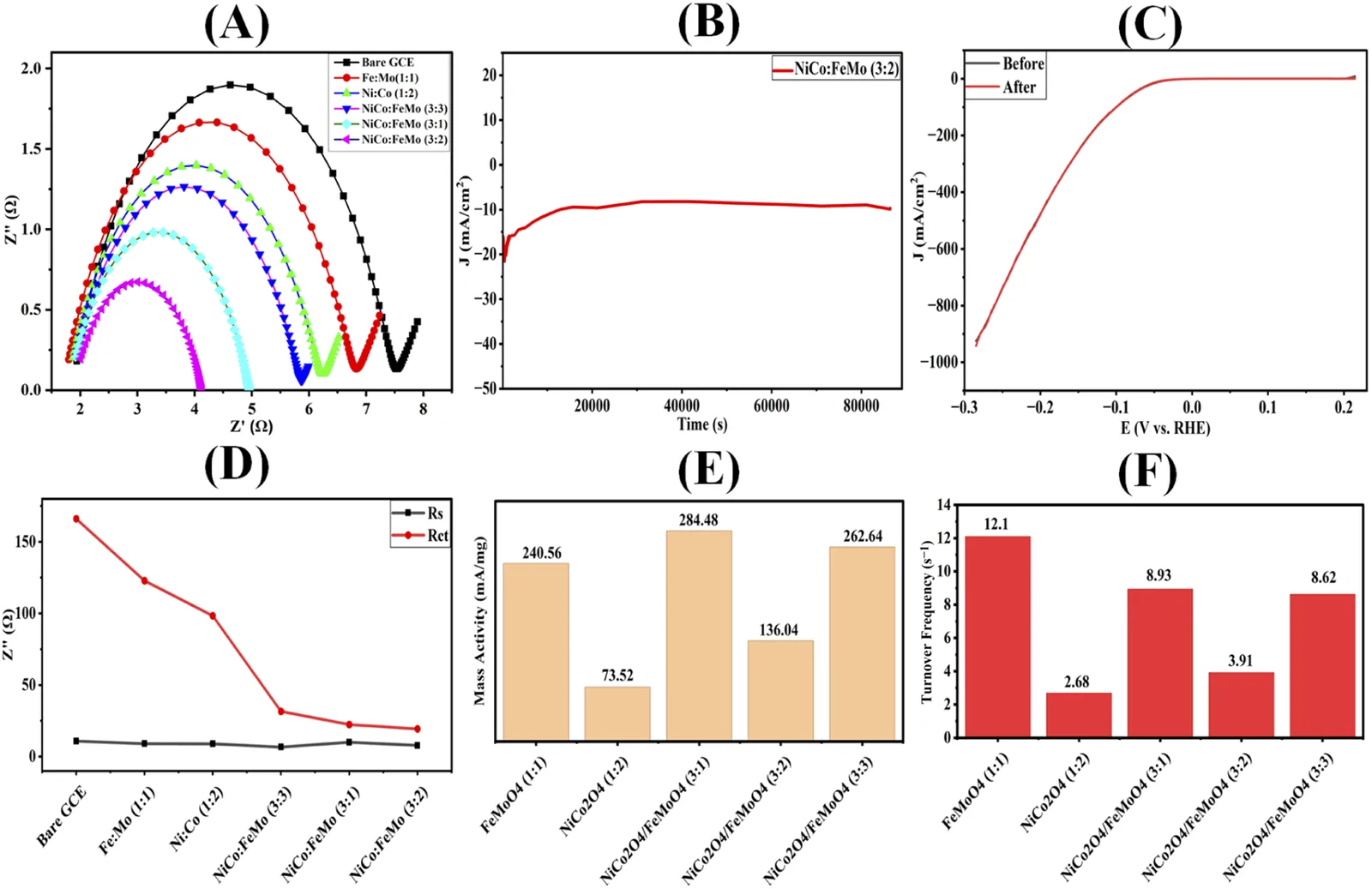
(A) EIS Nyquist plots of FeMoO_4_ (1 : 1), NiCo_2_O_4_ (1 : 2), NiCo_2_O_4_/FeMoO_4_ (3 : 1, 3 : 2, 3 : 3) in 0.5 M H_2_SO_4_, (B) chronoamperometric stability of NiCo_2_O_4_/FeMoO_4_ (3 : 2) at −0.50 V *vs.* Ag/AgCl in 0.5 M H_2_SO_4_, (C) LSV curves before and after the chronoamperometric stability test in 0.5 M H_2_SO_4_, (D) *R*_s_–*R*_ct_ relation of FeMoO_4_ (1 : 1), NiCo_2_O_4_ (1 : 2), NiCo_2_O_4_/FeMoO_4_ (3 : 1, 3 : 2, 3 : 3), (E) mass activity of FeMoO_4_ (1 : 1), NiCo_2_O_4_ (1 : 2), NiCo_2_O_4_/FeMoO_4_ (3 : 1, 3 : 2, 3 : 3), and (F) TOF of FeMoO_4_ (1 : 1), NiCo_2_O_4_ (1 : 2), NiCo_2_O_4_/FeMoO_4_ (3 : 1, 3 : 2, 3 : 3).

The intrinsic catalytic activity was further assessed from the turnover frequency (TOF), calculated at an assumed surface-active-site density of 1 × 10^−9^ mol cm^−2^ to approximate the number of hydrogen molecules generated per active site per second (Fig. 5(F)).^44^ The resulting values fall within the range commonly reported for transition-metal-based HER electrocatalysts (approximately 2.6–12.1 s^−1^): pristine NiCo_2_O_4_ gives the lowest TOF (2.68 s^−1^), consistent with comparatively sluggish intrinsic kinetics, whereas pristine FeMoO_4_ gives the highest (12.1 s^−1^), and the 3 : 1 and 3 : 3 composites fall in between at 8.93 and 8.62 s^−1^, respectively. The optimized NiCo_2_O_4_/FeMoO_4_ (3 : 2) composite gives a lower TOF (3.91 s^−1^) than pristine FeMoO_4_, a result that should not be read as reduced intrinsic activity. TOF depends directly on the assumed number of active sites, and because the calculation here treats every electrochemically accessible metal atom as catalytically active, the larger ECSA obtained for the 3 : 2 composite corresponds to a larger estimated active-site population; normalizing the same reaction rate over this larger population necessarily lowers the calculated TOF. As TOF and ECSA capture different, complementary aspects of catalytic behaviour apparent per-site turnover *versus* the density of accessible sites the two parameters are best interpreted together rather than in isolation. Read jointly with the lower overpotential, smaller Tafel slope, and higher current density obtained for the 3 : 2 composite, the comparatively modest TOF value reflects this normalization procedure rather than any loss of intrinsic activity, and indicates that heterostructure formation redistributes catalytic behaviour between active-site density and per-site turnover rather than simply increasing or decreasing it.

Mass activity, which normalizes the catalytic current to the 0.25 mg catalyst loading, follows a related but not identical trend (Fig. 5(E)). FeMoO_4_ delivers 240.56 mA mg^−1^ against 73.52 mA mg^−1^ for NiCo_2_O_4_, and the composites improve further on NiCo_2_O_4_, reaching 284.48 and 262.64 mA mg^−1^ for the 3 : 1 and 3 : 3 ratios, respectively, while the 3 : 2 composite gives 136.04 mA mg^−1^ lower than 3 : 1 and 3 : 3, but still well above pristine NiCo_2_O_4_.^45^ This pattern is consistent with the TOF trend discussed above: although the 3 : 1 and 3 : 3 compositions give a higher mass-normalized current response, the 3 : 2 composition offers the best overall balance among overpotential, Tafel slope, and mass activity, which together are more representative of its practical HER performance than any single metric considered on its own.

For a systematic comparison, the polarization data were also converted into Tafel plots (Fig. 4(B)); the resulting slopes, together with the overpotential, EIS, mass-activity, and TOF results discussed above, are summarized for all catalysts in Table 1.

Consistent with its low overpotential, the NiCo_2_O_4_/FeMoO_4_ (3 : 2) composite also gives the smallest Tafel slope of the series (35.9 mV dec^−1^), well below NiCo_2_O_4_ (57 mV dec^−1^), FeMoO_4_ (71 mV dec^−1^), and bare GCE (78 mV dec^−1^), with the relationship between Tafel slope and overpotential for all compositions summarized in Fig. 4(D). Such a low Tafel slope is characteristic of rapid HER kinetics and is consistent with a reaction proceeding predominantly *via* the Volmer–Tafel pathway, in which recombination of adsorbed hydrogen atoms is rate-limiting.^46^ The elementary steps of the HER process in acidic medium are outlined below (eqn (1)–(3)):

Volmer step (proton adsorption)

1 H^+^ + e^−^ + M* → M–H*

Tafel step (recombination)

2 M–H* + M–H* → H_2_ + 2M*

Heyrovsky step (electrochemical desorption)

3 M–H* + H^+^ + e^−^ → H_2_ + M*

The experimentally observed Tafel slope (∼35.9 mV dec^−1^) is close to the theoretical value of 30 mV dec^−1^ expected for a Volmer–Tafel-limited pathway, which supports the mechanistic assignment above and is consistent with favorable hydrogen adsorption/desorption kinetics at the composite surface.^47^

This favorable kinetic behaviour is corroborated by electrochemical impedance spectroscopy, performed to probe the charge-transfer characteristics of the electrocatalysts; the corresponding Nyquist plots are shown in Fig. 5(A).^48^ The NiCo_2_O_4_/FeMoO_4_ (3 : 2) composite gives the lowest charge-transfer resistance of the series (2.251 Ω), compared with 4.441 Ω for NiCo_2_O_4_, 5.068 Ω for FeMoO_4_, and 5.619 Ω for bare GCE, while the 3 : 1 and 3 : 3 composites fall at intermediate values of 3.155 and 4.037 Ω, respectively. This lower *R*_ct_ is consistent with faster interfacial charge transport at the composite electrodes, in line with the smaller Tafel slope discussed above; the corresponding *R*_s_–*R*_ct_ relationship for all catalysts is presented in Fig. 5(D). The full equivalent-circuit fitting parameters (CPE and Warburg diffusion element), together with the equivalent-circuit diagram used for the HER fits, are provided in Table S3 and Fig. S11 of the SI.

The operational durability of the best-performing NiCo_2_O_4_/FeMoO_4_ (3 : 2) composite was evaluated by chronoamperometry under HER conditions in 0.5 M H_2_SO_4_ at a fixed applied potential of −0.50 V *vs.* Ag/AgCl (Fig. 5(B)). Following an initial transient, the current density stabilized and remained approximately constant for the remainder of the test, which extended to approximately 24 h.^49^ This behaviour indicates that the composite maintains a comparatively stable electrochemical response under continuous HER operation, without the progressive current decay that would indicate significant catalyst degradation. Consistent with this, LSV curves recorded before and after the stability test (Fig. 5(C)) are closely superimposed across the measured potential range, with no discernible shift in the response to polarization. Together, these observations are consistent with good electrochemical durability of the composite under the HER conditions tested; whether this is also accompanied by structural or compositional stability of the catalyst would require post-stability characterization (*e.g.*, XRD, XPS) that was not performed in the present study.

Taken together, the low overpotential (40 mV), small Tafel slope (35.9 mV dec^−1^), low charge-transfer resistance (2.251 Ω), and enhanced mass activity of the NiCo_2_O_4_/FeMoO_4_ (3 : 2) composite point to a consistent improvement in HER performance relative to the individual oxide components an improvement that, combined with its OER performance (Section 3.3), points toward a promising bifunctional HER/OER electrocatalyst.

Relative to the single-phase catalysts tested here, the NiCo_2_O_4_/FeMoO_4_ composites give lower overpotentials and Tafel slopes across the series, consistent with the two components complementing rather than merely adding to one another. The porous morphology of the composite (Section 3.1) is expected to support electrolyte penetration and mass transport, in line with the higher ECSA and mass activity values obtained relative to the parent oxides, while the lower charge-transfer resistance obtained for both HER and OER (Table 1; Section 3.3) points to comparatively rapid interfacial kinetics on both electrodes. Overall, the bifunctional performance of the NiCo_2_O_4_/FeMoO_4_ (3 : 2) composite reflects a favorable combination of overpotential, Tafel slope, charge-transfer resistance, and active-site accessibility, as summarized in Table 1, with its OER behavior examined in detail in the following section, and a unified benchmarking of the catalyst's HER and OER performance against recently reported bifunctional electrocatalysts (2025–2026) presented in Section 3.5.

### 3.3 Catalytic performance for OER

OER activity of the as-prepared electrocatalysts was evaluated in 1.0 M KOH using GCE as the conductive substrate, combining LSV, Tafel analysis, EIS, and ECSA measurements to characterize catalytic activity, reaction kinetics, and interfacial charge-transfer behaviour. To optimize the Ni : Co ratio, a series of Ni–Co oxide catalysts with different Ni : Co ratios (3 : 1, 1 : 3, 2 : 1, 1 : 1, and 1 : 2), together with NiO, CoO, and bare GCE, were evaluated by LSV, Tafel analysis, and EIS (Fig. S5(A–C)) and (Table S2). Among these, the Ni : Co (1 : 2) composition gave the lowest overpotential (410 mV), outperforming both monometallic Ni (1 : 0, 530 mV) and Co (0 : 1, 430 mV) oxides. This improvement is consistent with literature reports that mixed Ni/Co oxides undergo *in situ* surface reconstruction to catalytically active NiOOH/CoOOH-type oxyhydroxide species during anodic polarization in alkaline media; such reconstruction was not independently verified in the present study and is noted here as a plausible, literature-supported interpretation rather than a directly demonstrated mechanism.

This trend is mirrored in the kinetic and interfacial behaviour of the series. Tafel analysis (Fig. S5(B)) gives the lowest slope for Ni : Co (1 : 2) among the compositions tested (112 mV dec^−1^), consistent with comparatively facile adsorption–desorption equilibria for the OH*, O*, and OOH* intermediates of the four-electron OER pathway. This composition also gives the largest double-layer capacitance (1.42 mF cm^−2^) in the ECSA analysis (Fig. S8), corresponding to an ECSA of 35.5 cm^2^ and a roughness factor of 507 (Table S2), consistent with a greater density of electrochemically accessible sites, and the lowest charge-transfer resistance (98.31 Ω) in the corresponding Nyquist plots (Fig. S5(C)), consistent with faster interfacial charge transfer. On this basis, the Ni : Co (1 : 2) composition was selected as the NiCo_2_O_4_ component for heterostructure construction with FeMoO_4_.

The intrinsic OER activity of FeMoO_4_ was also examined against bare GCE. In the LSV polarization curves (Fig. S5(D)), bare GCE requires a high overpotential of 870 mV to reach comparable current densities, reflecting its poor intrinsic OER activity, whereas FeMoO_4_ reaches similar current densities at a substantially lower overpotential of 570 mV, indicating appreciable intrinsic OER activity for this phase. This is accompanied by a markedly lower Tafel slope for FeMoO_4_ (93 mV dec^−1^) than for bare GCE (Fig. S5(E)), consistent with faster OER kinetics, and by a lower charge-transfer resistance in EIS measurements (122.8 Ω for FeMoO_4_*versus* 166.1 Ω for bare GCE; Fig. S5(F)), consistent with faster interfacial charge transfer. The OER activity of FeMoO_4_ is likely associated with its Fe^3+^ and Mo^6+^ redox centers, which are generally considered to provide additional sites for hydroxide adsorption during the OER.^50^

**Fig. 6:**
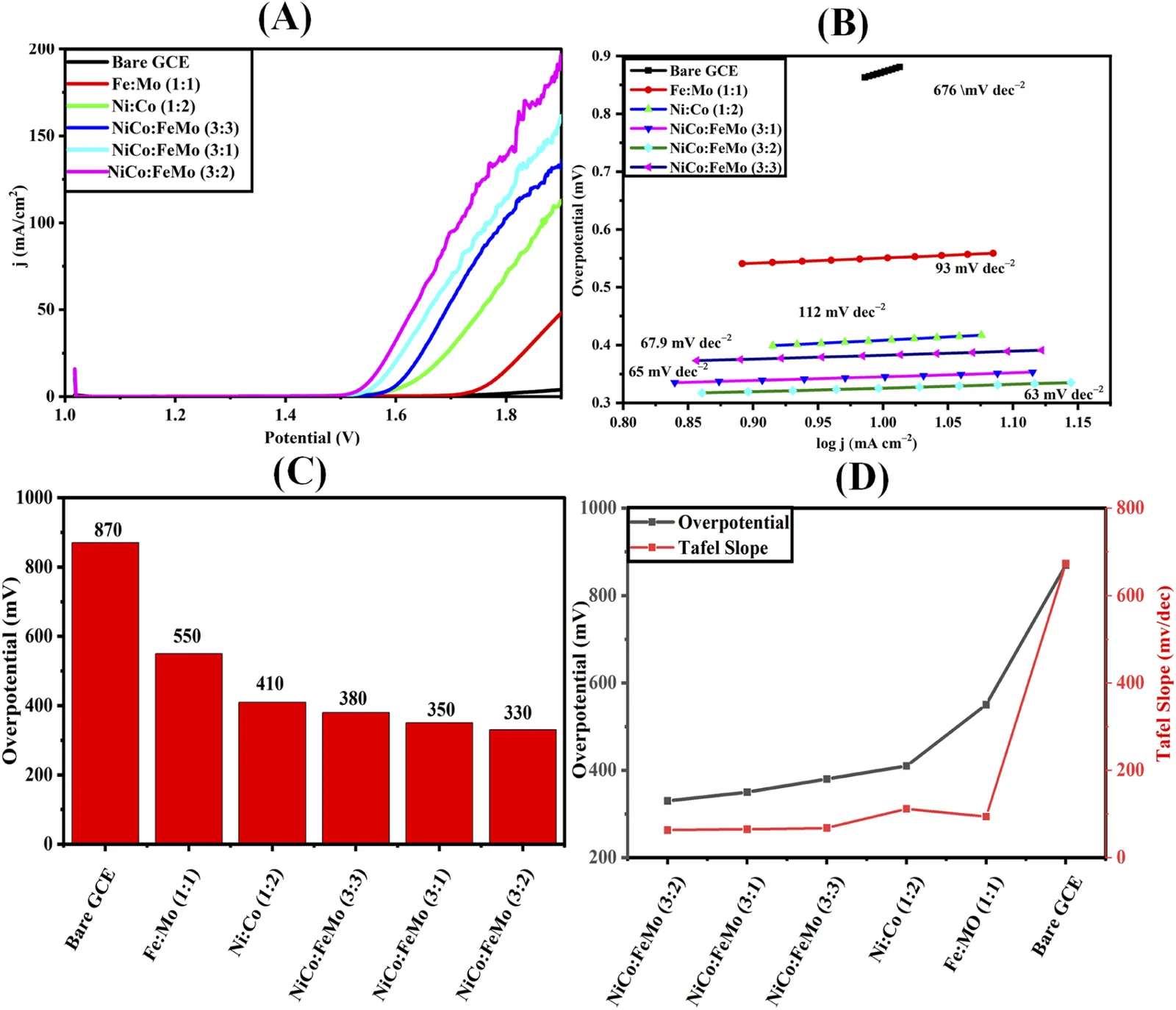
(A) Current–potential profiles from LSV measurements, (B) Tafel kinetic plots obtained in 1.0 M KOH at 10 mV s^−1^ scan rate, (C) benchmarking of overpotential values, and (D) relationship between Tafel slopes and overpotentials for bare GCE, FeMoO_4_ (1 : 1), NiCo_2_O_4_ (1 : 2), and NiCo_2_O_4_/FeMoO_4_ composites (3 : 1, 3 : 2, 3 : 3).

The full catalyst series was then compared by LSV in 1.0 M KOH (Fig. 6(A)) and by the corresponding Tafel analysis (Fig. 6(B)). Bare GCE shows the highest Tafel slope of the series (676 mV dec^−1^), consistent with its high overpotential and poor intrinsic OER activity, while FeMoO_4_ (93 mV dec^−1^) and NiCo_2_O_4_ (112 mV dec^−1^) give substantially lower values. The NiCo_2_O_4_/FeMoO_4_ composites lower the Tafel slope further still, to 65, 63, and 67.9 mV dec^−1^ for the 3 : 1, 3 : 2, and 3 : 3 ratios, respectively, pointing to faster OER kinetics across the composite series relative to either parent oxide.

Among the composites, Fig. 6(C) shows overpotentials of 350, 330, and 380 mV for the 3 : 1, 3 : 2, and 3 : 3 ratios, respectively, with the 3 : 2 composite giving the lowest overpotential of the series. This improvement over the individual components (NiCo_2_O_4_, FeMoO_4_) is consistent with a combined contribution of Ni, Co, Fe, and Mo active centers to the OER activity.

This trend parallels the Tafel slope results discussed above, where the heterostructured composites likewise outperform both parent oxides, with the NiCo_2_O_4_/FeMoO_4_ (3 : 2) composite giving the smallest Tafel slope of the series (63 mV dec^−1^) and thus the fastest apparent charge-transfer kinetics among the compositions tested. Fig. 6(D) summarizes the relationship between Tafel slope and overpotential for all the investigated catalysts.

**Fig. 7:**
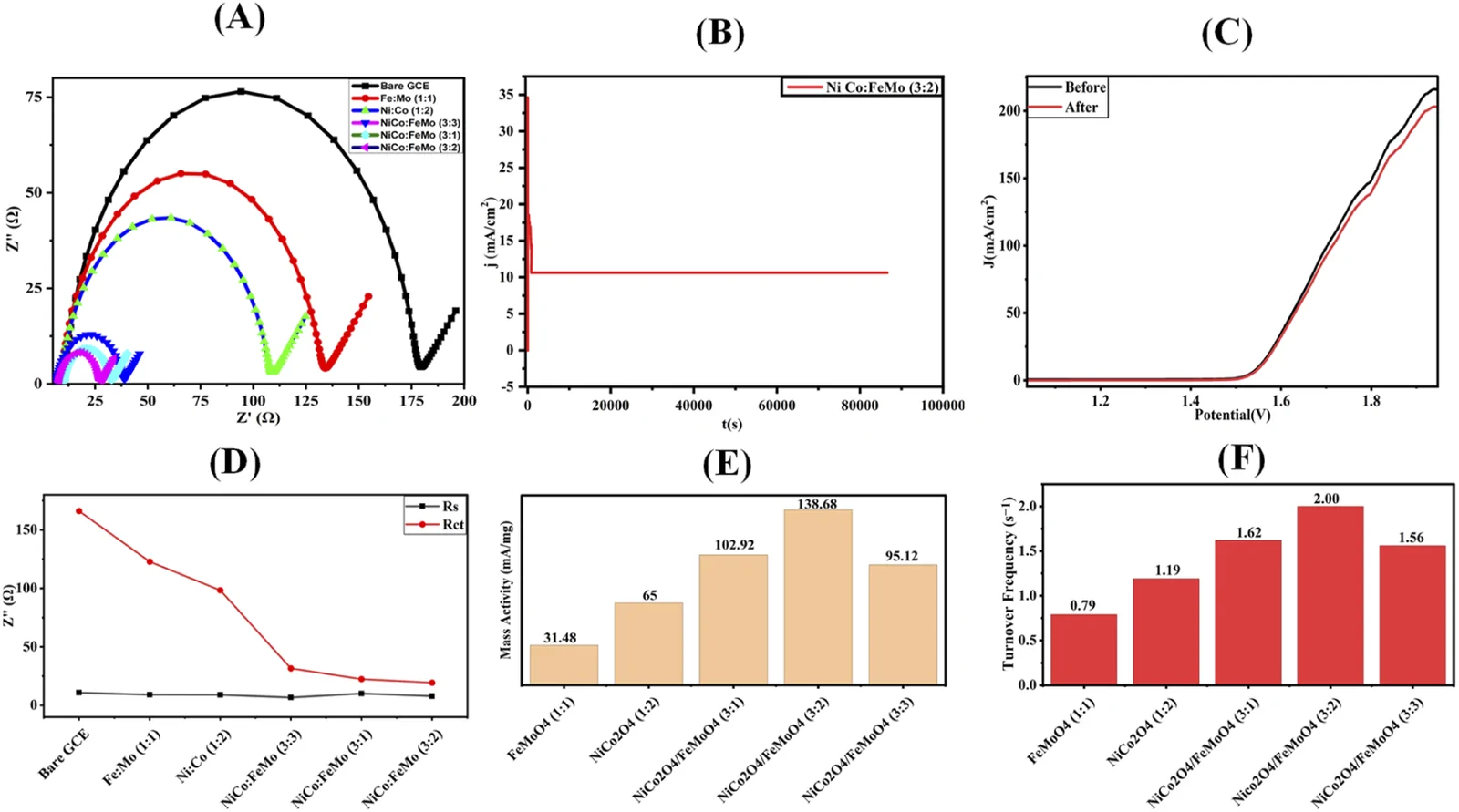
(A) EIS Nyquist plots of FeMoO_4_ (1 : 1), NiCo_2_O_4_ (1 : 2), NiCo_2_O_4_/FeMoO_4_ (3 : 1, 3 : 2, 3 : 3) in 1 M KOH, (B) chronoamperometry of NiCo_2_O_4_/FeMoO_4_ at +1.50 V *vs.* Ag/AgCl in 1.0 M KOH (3 : 2), (C) post-stability LSV in 1.0 M KOH at a scan rate of 10 mV s^−1^, (D) *R*_s_–*R*_ct_ relation of FeMoO_4_ (1 : 1), NiCo_2_O_4_ (1 : 2), NiCo_2_O_4_/FeMoO_4_ (3 : 1, 3 : 2, 3 : 3), (E) mass activity of FeMoO_4_ (1 : 1), NiCo_2_O_4_ (1 : 2), NiCo_2_O_4_/FeMoO_4_ (3 : 1, 3 : 2, 3 : 3) and (F) TOF of FeMoO_4_ (1 : 1), NiCo_2_O_4_ (1 : 2), NiCo_2_O_4_/FeMoO_4_ (3 : 1, 3 : 2, 3 : 3).

Electrode–electrolyte charge-transfer behavior was probed by EIS, with the corresponding Nyquist plots shown in Fig. 7(A). The *R*_s_ values are closely similar across all catalysts, indicating comparable electrolyte resistance, whereas *R*_ct_ decreases markedly upon heterostructure formation: from 166.1 Ω for bare GCE to 122.8 Ω for FeMoO_4_ (1 : 1) and 98.31 Ω for NiCo_2_O_4_ (1 : 2), and further to 22.33, 19.31, and 31.54 Ω for the 3 : 1, 3 : 2, and 3 : 3 composites, respectively, with the 3 : 2 composite giving the lowest value of the series. This trend is consistent with progressively faster interfacial charge transfer as the heterostructure is formed, in line with the corresponding decrease in Tafel slope discussed above; the *R*_s_–*R*_ct_ relationship for all catalysts is summarized in Fig. 7(D). The full equivalent-circuit fitting parameters (CPE and Warburg diffusion element), together with the equivalent-circuit diagram used for the OER fits, are provided in Table S4 and Fig. S12 of the SI.

The durability of the NiCo_2_O_4_/FeMoO_4_ (3 : 2) composite was assessed by chronoamperometry at a fixed applied potential of +1.50 V *vs.* Ag/AgCl (Fig. 7(B)), which shows only a slight decline in current over extended operation, and by LSV recorded before and after this test (Fig. 7(C)), where the post-stability curve is closely superimposed on the initial one with only a minor shift. Together, these observations are consistent with stable electrochemical performance under the alkaline OER conditions tested; whether this behaviour is also accompanied by structural or compositional stability of the catalyst would require post-stability characterization (*e.g.*, XRD, XPS) that was not performed in the present study.

ECSA was estimated from the double-layer capacitance (*C*_dl_) obtained by CV. FeMoO_4_ (1 : 1) gives a *C*_dl_ of 1.03 mF cm^−2^ (ECSA = 25.75 cm^2^), and NiCo_2_O_4_ (1 : 2) a larger value of 1.42 mF cm^−2^ (ECSA = 35.50 cm^2^) (Fig. S9), while the NiCo_2_O_4_/FeMoO_4_ composites give still larger ECSA values (Fig. S10): *C*_dl_ of 1.65, 1.80, and 1.58 mF cm^−2^, corresponding to ECSA values of 41.25, 45.0, and 39.5 cm^2^ for the 3 : 1, 3 : 2, and 3 : 3 ratios, respectively. The largest ECSA, obtained for the 3 : 2 composite, is consistent with a greater density of electrochemically accessible active sites, which may contribute to its improved OER performance.

The same trend carries through to current density at fixed potential: FeMoO_4_ (1 : 1) and NiCo_2_O_4_ (1 : 2) reach 110.80 and 228.87 mA cm^−2^, respectively, while the NiCo_2_O_4_/FeMoO_4_ composites reach substantially higher values of 362.39, 488.31, and 334.93 mA cm^−2^ for the 3 : 1, 3 : 2, and 3 : 3 ratios. Table 2 collects these overpotential, Tafel slope, EIS, ECSA, and current-density results for all catalysts, and shows that the composite electrocatalysts consistently combine faster OER kinetics (lower Tafel slopes) and lower overpotentials than bare GCE, consistent with the greater active-site availability reflected in the ECSA data above.

**Table 2:** Electrochemical parameters obtained from measurements at bare GCE and GCE modified with Ni : Co (1 : 2), FeMoO_4_, and NiCo_2_O_4_/FeMoO_4_ composites for OER performance evaluation

Electrocatalyst	*η*_10_ (mV)	Tafel slope (mV dec^−1^)	*R*_s_ (Ω)	*R*_ct_ (Ω)	Current density (mA cm^−2^)	*C*_dl_ (mF cm^−2^)	ECSA (cm^2^)	RF	Mass activity (mA mg^−1^)	TOF (s^−1^)
Bare GCE	870	676	10.73	166.1	—	—	—	—	—	—
FeMoO_4_ (1 : 1)	570	93	9.0	122.8	110.80	1.03	25.75	368	31.48	0.79
NiCo_2_O_4_ (1 : 2)	410	112	8.9	98.31	228.87	1.42	35.50	507	65.00	1.19
NiCo_2_O_4_/FeMoO_4_ (3 : 1)	350	65	9.98	22.33	362.39	1.65	41.25	589	102.92	1.62
NiCo_2_O_4_/FeMoO_4_ (3 : 2)	330	63	7.8	19.31	488.31	1.80	45.00	643	138.68	2.00
NiCo_2_O_4_/FeMoO_4_ (3 : 3)	380	67.9	6.64	31.54	334.93	1.58	39.50	564	95.12	1.56

Mass activity, normalized to a 0.25 mg catalyst loading, shows the same overall pattern (Fig. 7(E)): the NiCo_2_O_4_/FeMoO_4_ (3 : 2) composite gives the highest value of the series (138.68 mA mg^−1^), well above NiCo_2_O_4_ (65.00 mA mg^−1^) and FeMoO_4_ (31.48 mA mg^−1^), while the 3 : 1 and 3 : 3 composites give intermediate values of 102.92 and 95.12 mA mg^−1^, respectively. This trend points to more efficient utilization of the active material following heterostructure formation.

Turnover frequency was also evaluated for OER, using the same assumed surface-active-site density of 1 × 10^−9^ mol cm^−2^ applied for HER (Section 3.2), based on the ECSA data above. TOF values across the series range from 0.79 to 2.00 s^−1^ (Fig. 7(F)), with the NiCo_2_O_4_/FeMoO_4_ (3 : 2) composite giving the highest value (2.00 s^−1^). As discussed for HER, this apparent TOF reflects both the intrinsic activity of the catalyst and the assumed number of active sites, and is best interpreted alongside the ECSA and mass-activity results rather than in isolation; considered together, these parameters indicate that heterostructure formation improves both the density of accessible active sites and their per-site catalytic efficiency for OER.

Taken together, the lower overpotential (330 mV), smaller Tafel slope (63 mV dec^−1^), lower charge-transfer resistance (19.31 Ω), higher current density (488.31 mA cm^−2^), and larger ECSA (45 cm^2^) obtained for the NiCo_2_O_4_/FeMoO_4_ (3 : 2) composite are consistent with a combined contribution of the NiCo_2_O_4_ and FeMoO_4_ phases to its OER activity; establishing the underlying interfacial or electronic origin of this improvement would require characterization beyond the scope of the present study. Its bifunctional HER/OER performance is benchmarked against recently reported literature catalysts in Section 3.5.

### 3.4 Proposed mechanism of OER

With the mechanistic picture necessarily constrained by the macroscopic, ensemble-averaged nature of the electrochemical measurements above, the improved OER activity of the NiCo_2_O_4_/FeMoO_4_ composite is consistent with the coexistence of the conductive spinel NiCo_2_O_4_ phase and the redox-active FeMoO_4_ phase on the same electrode, together with the lower charge-transfer resistance and larger ECSA measured for this composite relative to the individual phases (Section 3.3). In alkaline electrochemical processes, the catalyst surface is generally reported to undergo partial surface reconstruction, producing oxyhydroxide species such as NiOOH and CoOOH that are widely proposed to act as active sites for oxygen evolution; this reconstruction was not independently verified in the present study. The presence of FeMoO_4_ alongside NiCo_2_O_4_ introduces additional Fe- and Mo-based redox centers at the electrode surface, which may contribute to the improved OER activity of the composite; confirming whether this reflects a genuine electronic interaction between the two phases would require further characterization beyond the scope of the present study.

In alkaline electrolyte, the OER occurs *via* a four-electron transfer mechanism that entails the adsorption and transformation of hydroxide ions on the catalyst surface. The fundamental reaction steps can be articulated as follows:

• Hydroxide adsorption* + OH^−^ → *OH + e^−^

• Formation of adsorbed oxygen species*OH + OH^−^ → O* + H_2_O + e^−^

• Formation of hydroperoxo intermediateO* + OH^−^ → OOH* + e^−^

• Oxygen evolution and regeneration of reactive surface regionsOOH* + OH^−^ → * + H_2_O + O_2_ + e^−^* represents the reactive surface regions or active sites. The lower charge-transfer resistance and larger ECSA measured for the NiCo_2_O_4_/FeMoO_4_ composite relative to the individual phases (Section 3.3) are consistent with more efficient charge transfer and a greater number of accessible active sites for adsorption/desorption of reaction intermediates, which may contribute to a lower effective activation barrier for oxygen evolution. Moreover, the coexistence of multiple redox-active centers (Ni^2+^/Ni^3+^, Co^2+^/Co^3+^, Fe^2+^/Fe^3+^, and Mo^6+^) in the composite may contribute to the enhanced OER activity by providing additional pathways for charge transfer. The hierarchical porous structure is also expected to enhance electrolyte infiltration and increase the availability of electrochemically active sites, consistent with the improved OER performance observed for the composite.

**Fig. 8:**
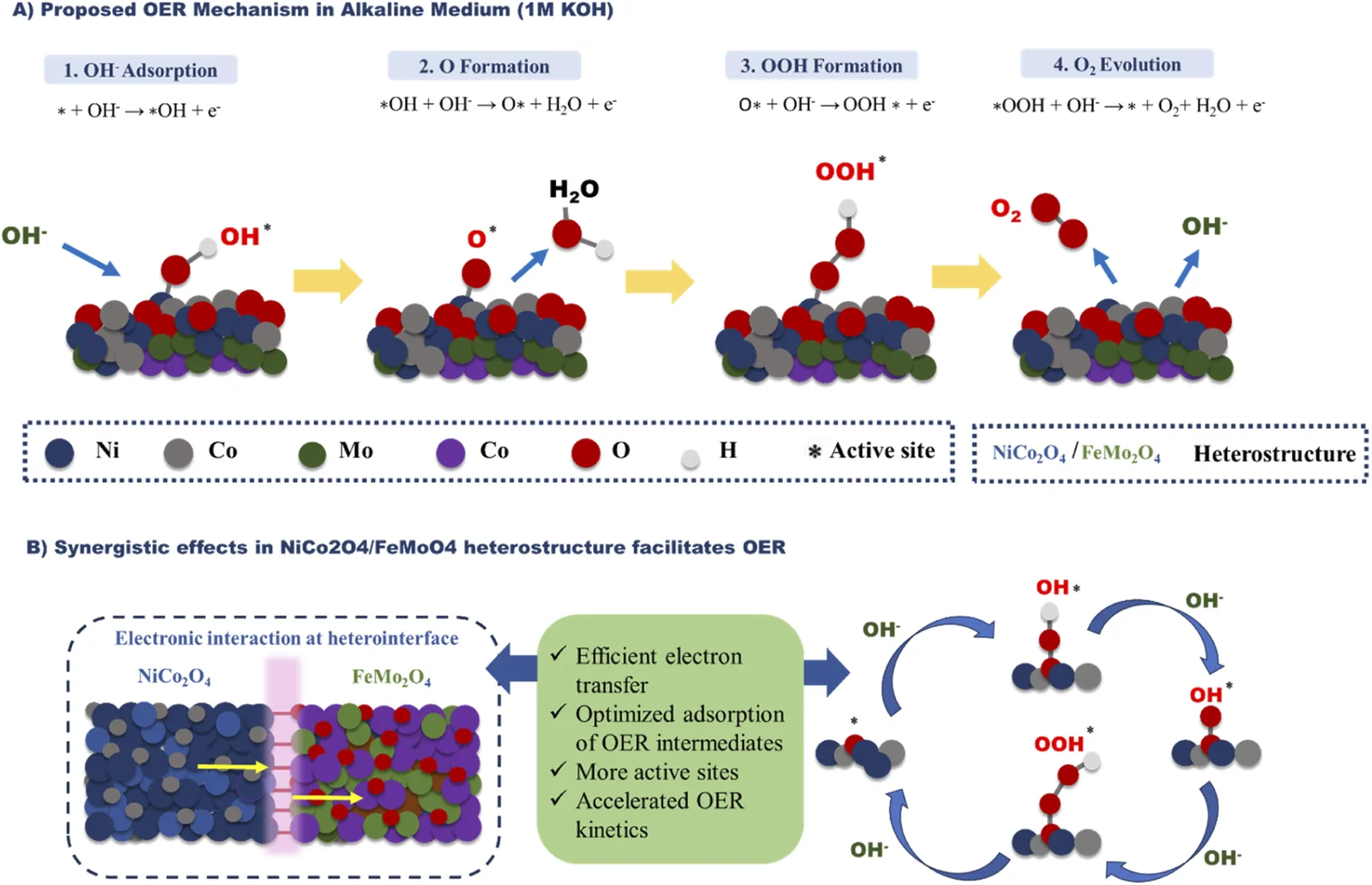
Proposed OER mechanism for NiCo_2_O_4_/FeMoO_4_ heterostructured electrocatalyst in alkaline medium.

It should be noted that the mechanistic discussion above is based solely on macroscopic electrochemical measurements (overpotential, EIS, ECSA) and on compositional evidence for the coexistence of NiCo_2_O_4_ and FeMoO_4_ rather than on direct nanoscale structural evidence from HRTEM (Fig. 8).

### 3.5 Benchmarking against recently reported bifunctional HER/OER electrocatalysts

Because practical water electrolysis requires a single catalyst to sustain both half-reactions, evaluating HER and OER activity in isolation gives an incomplete picture of a material's usefulness for overall water splitting. Two catalysts with comparable HER overpotentials can differ substantially in their OER behavior, and *vice versa*, so a meaningful assessment of bifunctional viability requires that both metrics be considered together, alongside the electrolyte, substrate, and current density at which they were measured, since these strongly influence the reported values. Therefore, Table 3 presents the HER and OER performance of the NiCo_2_O_4_/FeMoO_4_ (3 : 2) composite alongside recently reported (2025–2026) bifunctional electrocatalysts, compared simultaneously on both reactions rather than through separate HER and OER benchmarking tables.

**Table 3:** Comparison of the bifunctional HER and OER performance of the NiCo_2_O_4_/FeMoO_4_ (3 : 2) composite with recently reported bifunctional electrocatalysts

Electrocatalysts	*η*_10_ for OER (mV)	Tafel slope (OER)	*η*_10_ for HER (mV)	Tafel slope (HER)	Substrate	Electrolyte (OER)	Electrolyte (HER)	Ref.
FeOCl/g-C_3_N_4_	430	74	235	86.26	GCE	1 M KOH	0.5 M H_2_SO_4_	51
HEMO–CeO_2_	380	64	223	114	NF	1 M KOH	1 M KOH	52
NiCo_2_O_4_@CoMoS	374.4@100 mA cm^−2^	175.47	314.6@50 mA cm^−2^	70.9	NF	1 M KOH	1 M KOH	53
CC/NiMo@Co–NSC	335@50 mA cm^−2^	—	66	65	CC	0.5 M H_2_SO_4_	0.5 M H_2_SO_4_	54
CoBiWO_4_	180	115	370	144	NF	2 M KOH	0.5 M H_2_SO_4_	55
Co–Ba–TPA MOF	412.5	106.18	414.5	145.23	GCE	1 M KOH	1 M KOH	56
BMNi/Co-NP	440	71	270	82.9	GCE	1 M KOH	0.5 M H_2_SO_4_	57
TiCoCuMoNi	400@100 mA cm^−2^	173.45	35@100 mA cm^−2^	51.32	Self-supported	1 M KOH	1 M KOH	58
Fe-doped SrCoO_3_	400	58.62	379	58.62	GCE	1 M KOH	1 M KOH	59
Ni–PTC MOF	400	150.7	241	72.5	NF	3 M KOH	3 M KOH	60
NiSe_2_/CoSe_2_	400@500 mA cm^−2^	49	342@500 mA cm^−2^	∼85	NF	1 M KOH	1 M KOH	61
**NiCo**_**2**_ **O**_**4**_ **/FeMoO**_**4**_ **(3 : 3)**	380	67.9	70	36.8	GCE	1 M KOH	0.5 M H_2_SO_4_	**This work**
**NiCo**_**2**_ **O**_**4**_ **/FeMoO**_**4**_ **(3 : 1)**	350	65	60	37.5	GCE	1 M KOH	0.5 M H_2_SO_4_	**This work**
**NiCo**_**2**_ **O**_**4**_ **/FeMoO**_**4**_ **(3 : 2)**	330	63	40	35.9	GCE	1 M KOH	0.5 M H_2_SO_4_	**This work**

Considered jointly rather than as separate half-reactions, the NiCo_2_O_4_/FeMoO_4_ (3 : 2) heterostructure occupies a competitive position among the compiled bifunctional catalysts in Table 3. Its HER overpotential of 40 mV (0.5 M H_2_SO_4_) approaches the lowest reported value, 35 mV for TiCoCuMoNi, though the latter was measured at a higher current density (100 mA cm^−2^) on a self-supported electrode; the composite's HER Tafel slope of 35.9 mV dec^−1^ is nevertheless lower than every value listed, consistent with comparatively fast interfacial kinetics. For OER (1.0 M KOH), only CoBiWO_4_ achieves a lower overpotential than the present catalyst (180 *vs.* 330 mV), while the remaining systems require 334–440 mV; its OER Tafel slope of 63 mV dec^−1^ is, however, exceeded by NiSe_2_/CoSe_2_ (49 mV dec^−1^) and Fe-doped SrCoO_3_ (58.62 mV dec^−1^), indicating that OER charge-transfer kinetics are not the strongest attribute of this system relative to the literature. These comparisons are constrained by differences in current density (10 *vs.* 50–500 mA cm^−2^), substrate (GCE *vs.* nickel foam or self-supported electrodes), and electrolyte, since several literature catalysts were evaluated for both reactions in a single alkaline medium whereas the present catalyst was tested in separate acidic and alkaline electrolytes. Within these constraints, the composite pairs a low HER overpotential and the lowest HER Tafel slope in the set with an OER overpotential surpassed only by CoBiWO_4_, supporting its potential as a low-cost bifunctional candidate, while single-electrolyte, device-relevant testing remains necessary to place it on a fully consistent footing with the recent literature.

## 4. Conclusions

A NiCo_2_O_4_/FeMoO_4_ heterostructured electrocatalyst was successfully synthesized *via* a hydrothermal route, with phase coexistence confirmed by XRD, FTIR, SEM-EDS, and XPS. The optimized 3 : 2 composite exhibited a porous morphology, higher ECSA, and lower charge-transfer resistance than the individual oxides, resulting in enhanced bifunctional electrocatalytic performance. For HER in 0.5 M H_2_SO_4_, it required only 40 mV overpotential at 10 mA cm^−2^ with a Tafel slope of 35.9 mV dec^−1^ and a TOF of 3.91 s^−1^. For OER in 1.0 M KOH, it delivered an overpotential of 330 mV, a Tafel slope of 63 mV dec^−1^, a mass activity of 138.68 mA mg^−1^, and a TOF of 2.00 s^−1^, together with good electrochemical stability. The superior performance relative to the parent oxides indicates a beneficial synergistic effect between NiCo_2_O_4_ and FeMoO_4_. Although direct atomic-scale characterization of the heterointerface and full-cell water-splitting evaluation remain necessary, these results demonstrate the potential of NiCo_2_O_4_/FeMoO_4_ heterostructures as low-cost, non-precious bifunctional electrocatalysts for water splitting.

## Conflicts of interest

The authors declare no competing interests.

## Supplementary Material

RA-016-D6RA06110A-s001

## Data Availability

The data supporting this article have been included as part of the supplementary information (SI). Supplementary information: tables and figures. See DOI: https://doi.org/10.1039/d6ra06110a.
