# Supplementary material for: NiCo2O4/FeMoO4 heterostructure as a bifunctional electrocatalyst for hydrogen and oxygen evolution reactions
Source: RSC Adv. 2026 Aug 19;16(41):48996–9011. doi: 10.1039/d6ra06110a (PMC13488453; doi:10.1039/d6ra06110a)
Supplement: Supplementary file 1 [file RA-016-D6RA06110A-s001.pdf]

Supplementary Information (SI) for RSC Advances.
This journal is © The Royal Society of Chemistry 2026

Supporting Document

$NiCo_2O_4/FeMoO_4$ Heterostructure as a Bifunctional Electrocatalyst for Hydrogen and Oxygen Evolution Reactions

Muhammad Tahir[1], Mohsin Javed[2], Suniya Shahzad[1], Shahid Iqbal[2], Mustafa Tuzen[3], Afzal Shah[1]*

[1]Department of Chemistry Quaid-i-Azam University, Islamabad 45320, Pakistan

[2]Department of Chemical and Environmental Engineering, University of Nottingham Ningbo, China, Ningbo 315100, China

[3]Tokat Gaziosmanpasa University, Faculty of Science and Arts, Chemistry Department, 60250 Tokat, Turkey

Correspondence*: afzals_qau@yahoo.com (Afzal Shah)

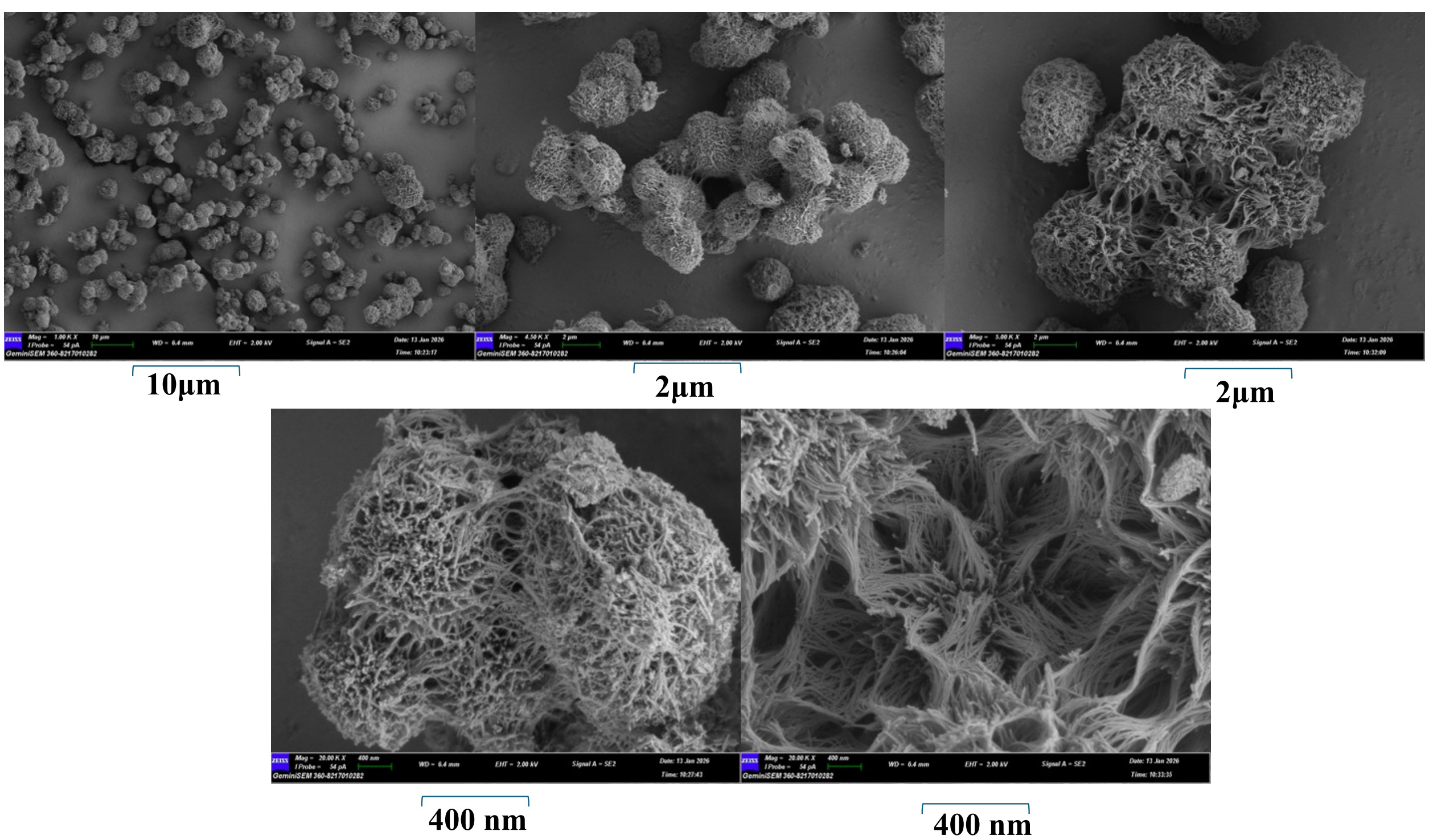

Figure S1: SEM images of $NiCo_2O_4$

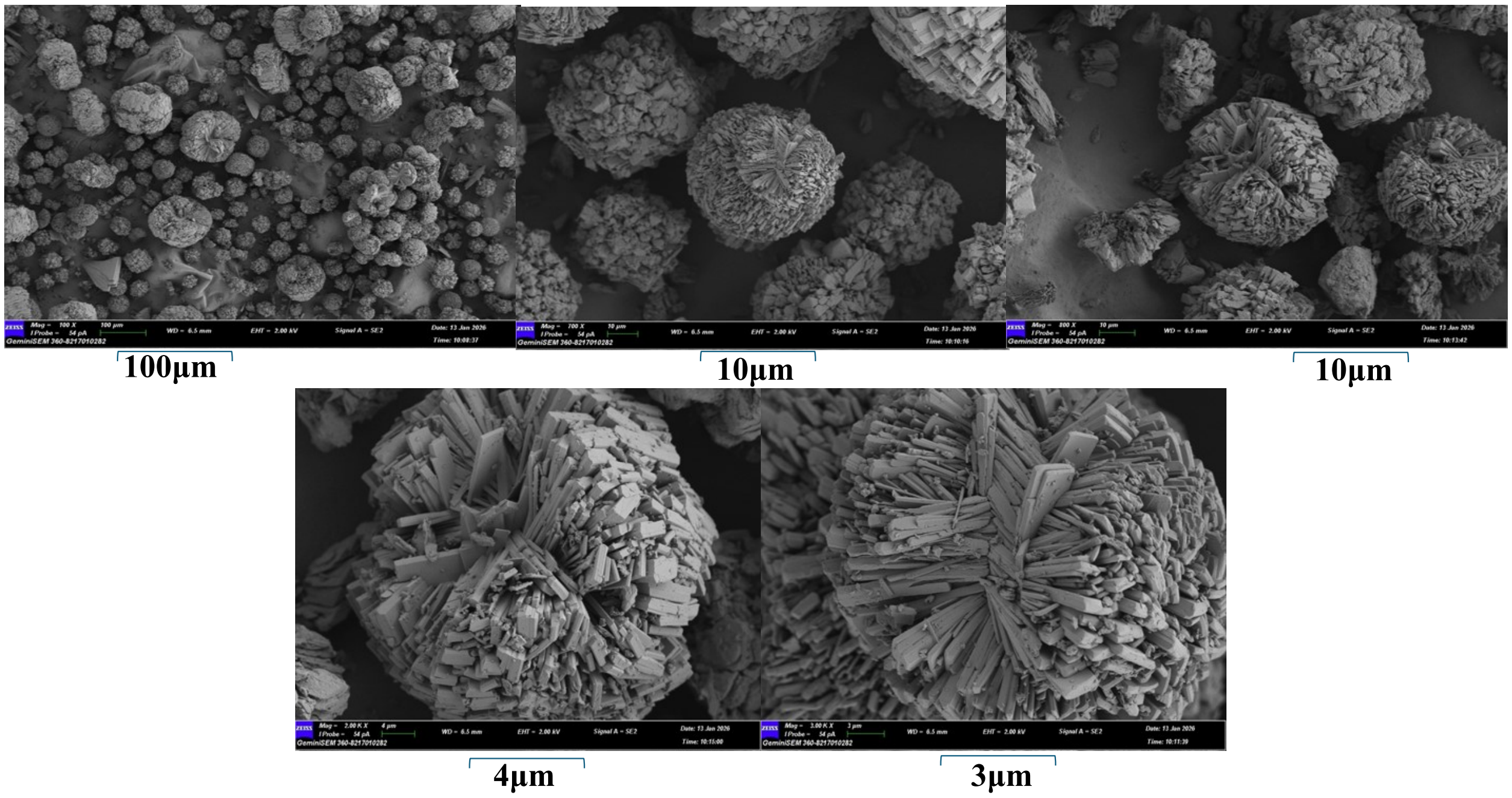

Figure S2: SEM images of $FeMoO_4$

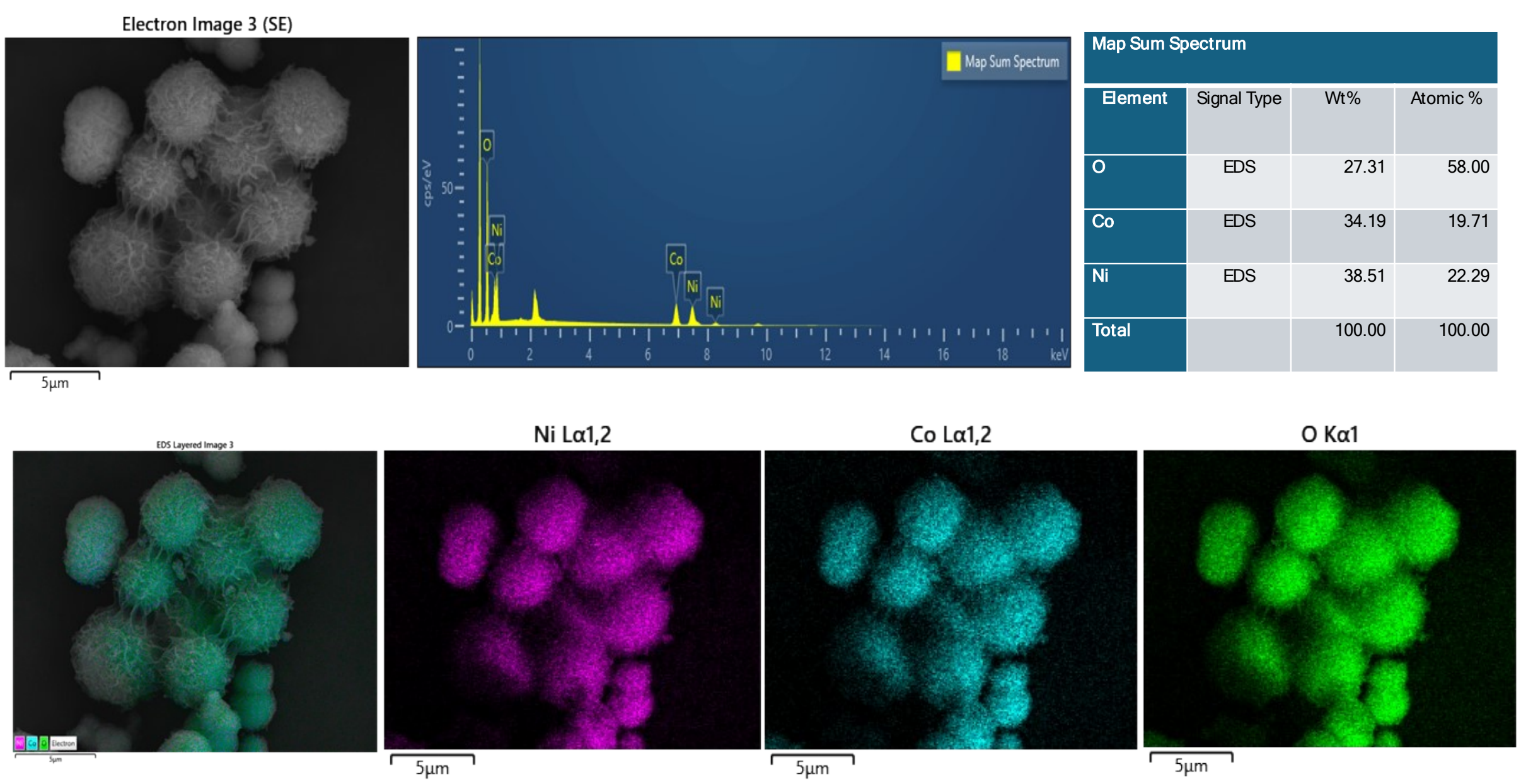

Map Sum Spectrum

Element	Signal Type	Wt%	Atomic %
O	EDS	27.31	58.00
Co	EDS	34.19	19.71
Ni	EDS	38.51	22.29
Total		100.00	100.00

Figure S3: EDS Analysis of $NiCo_2O_4$

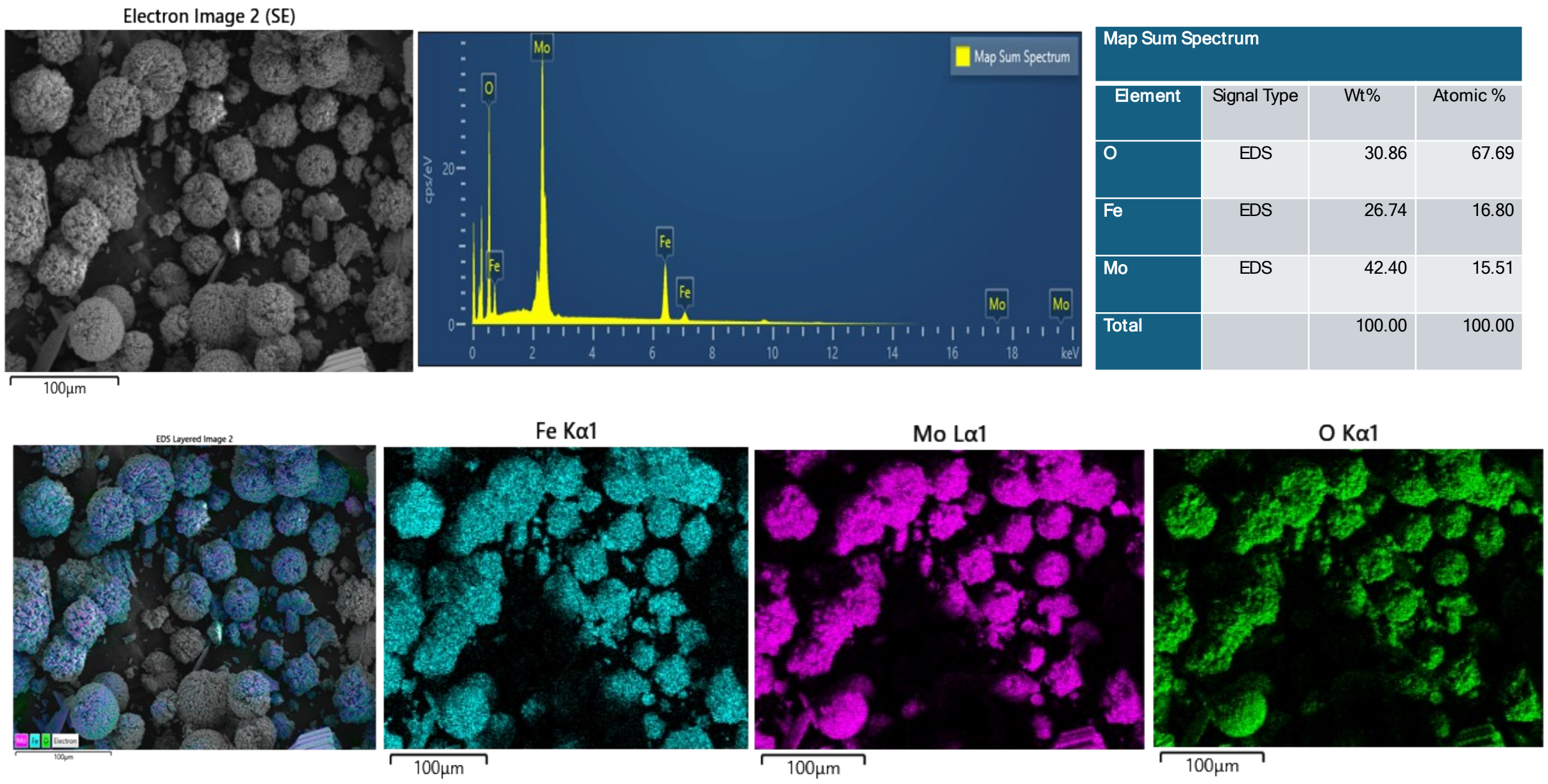

Figure S4: EDS Analysis of $FeMoO_4$

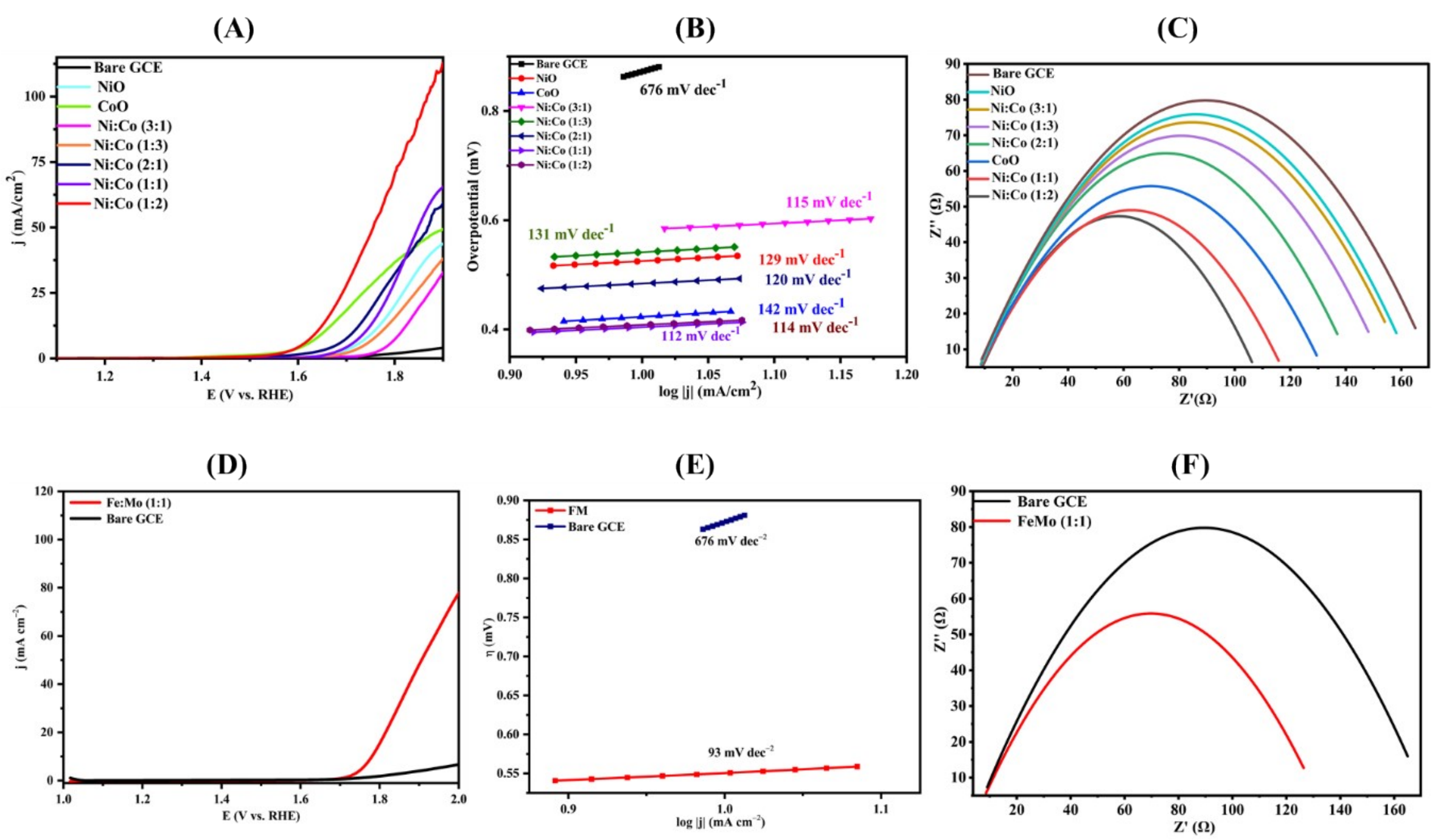

Figure S5: (A) LSV polarization curves, (B) Tafel plots, and (C) EIS spectra of the Ni-Co oxide series, including NiO (1:0), CoO (0:1), Ni-Co (3:1, 1:3, 2:1, 1:1, and 1:2), and bare GCE; (D) LSV polarization curve, (E) Tafel plot, and (F) EIS spectrum of $FeMoO_4$ and bare GCE in 1 M KOH.

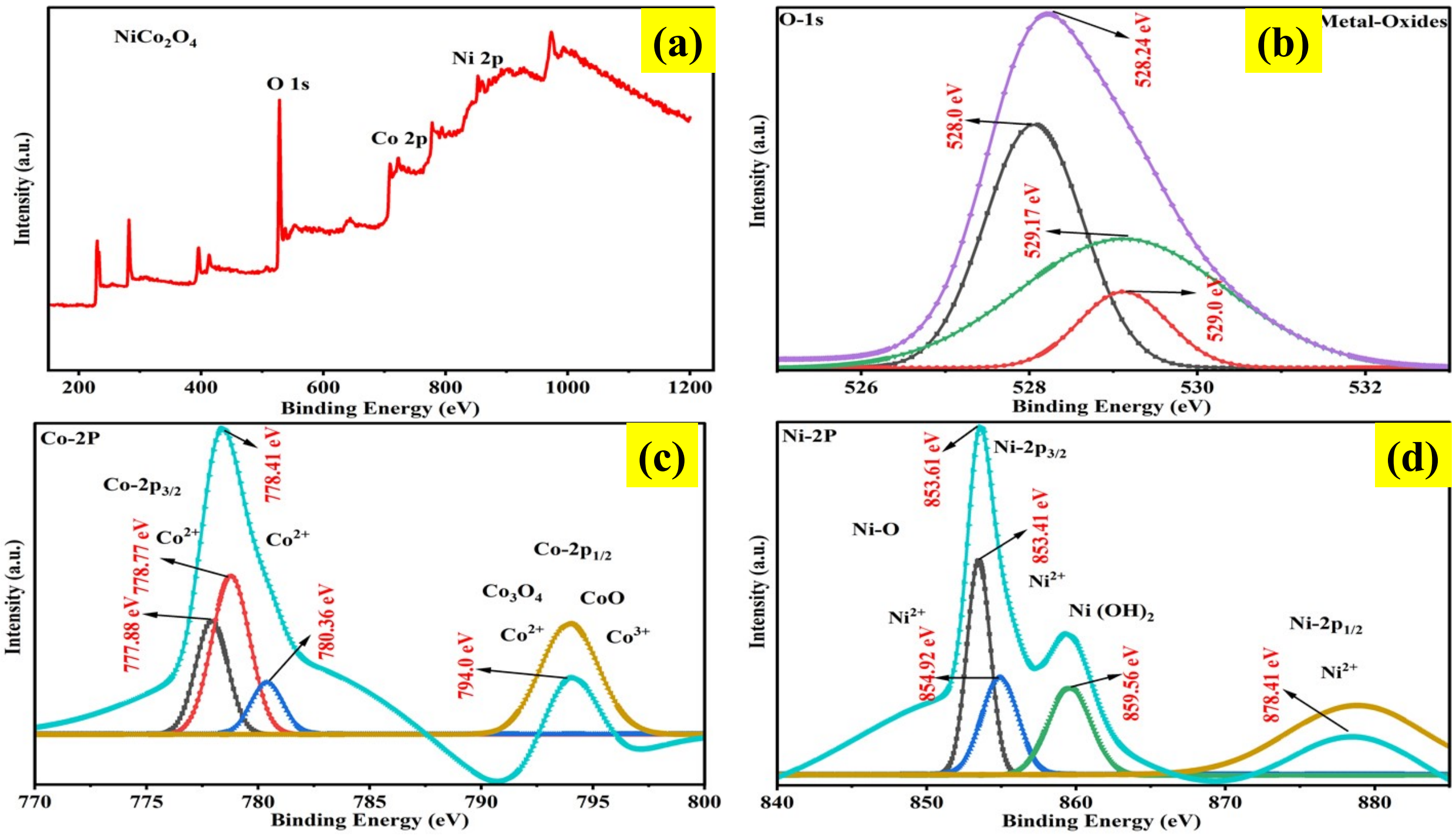

Figure S6: XPS Analysis of $NiCo_2O_4$.

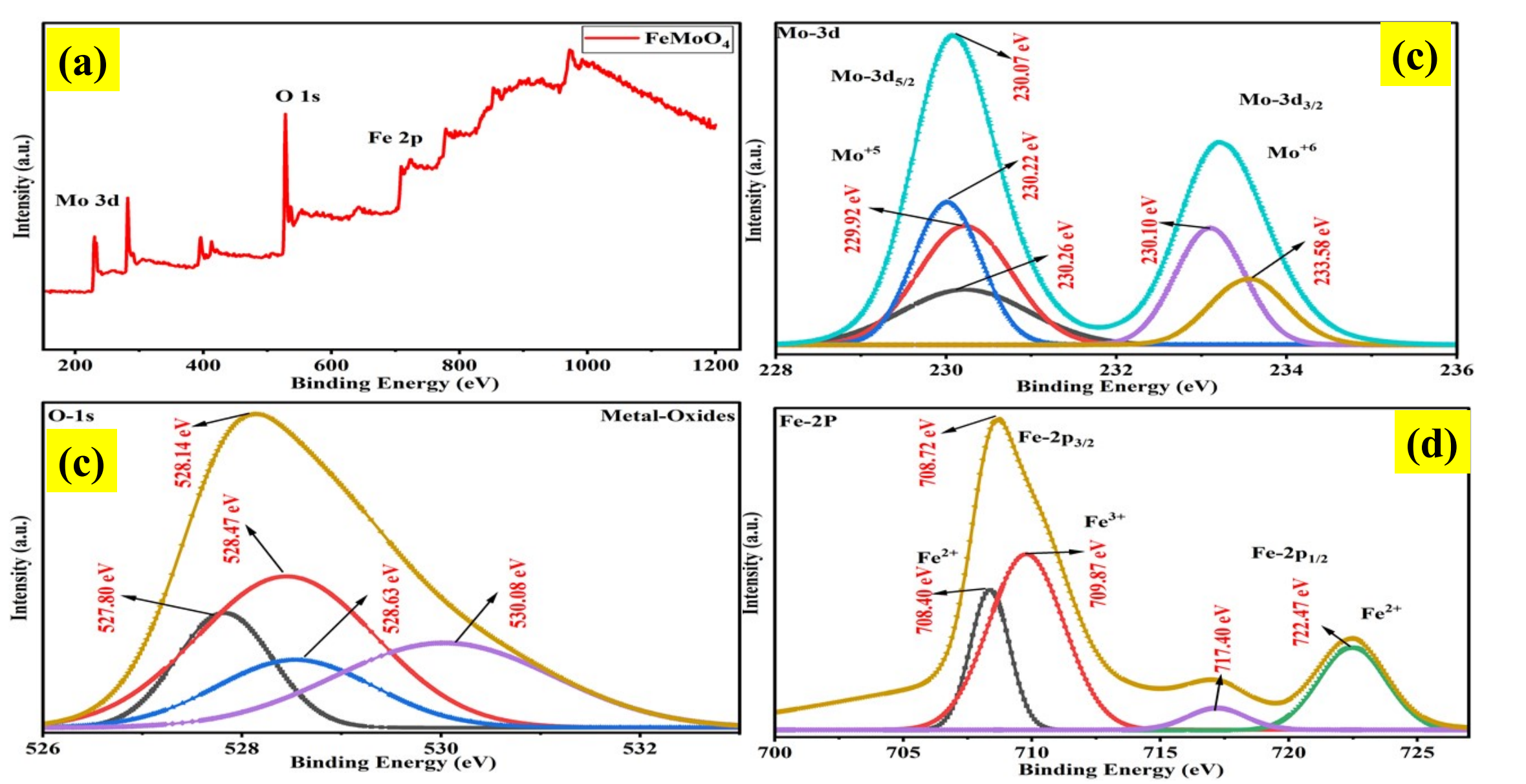

Figure S7: XPS Analysis of $FeMoO_4$.

Table S1. Comparison of XPS binding energies of pristine $NiCo_2O_4$, pristine $FeMoO_4$, and $NiCo_2O_4/FeMoO_4$ heterostructure.

Element	Peak Assignment	Pure Oxide (eV)	Composite (eV)	ΔBE (eV)
**Ni**	Ni $2p_{3/2}$	853.61	853.88	+0.27
**Ni**	Ni $2p_{1/2}$	878.41	878.32	−0.09
**Co**	Co $2p_{3/2}$	778.41	778.88	+0.47
**Fe**	Fe $2p_{3/2}$	708.72	709.40	+0.68
**Fe**	Fe $2p_{1/2}$	722.47	723.10	+0.63
**Mo**	Mo $3d_{5/2}$	230.07	230.58	+0.51
**Mo**	Mo $3d_{3/2}$	233.58	233.52	−0.06

Table S2: Electrochemical Parameters of Ni-Co Series

No	Electrocatalyst	Overpotential (mV)	Tafel Slope (mV dec^{-1})	Rs(Ω)	Rct (Ω)	C_{dl} (mF/cm^2)	ESCA (cm^2)	RF
**a**	Ni:Co (1:0)	530	129	8.9	148.8	0.45	11.25	161
**b**	Ni:Co (0:1)	430	142	9.1	117.8	1.38	34.5	493
**c**	Ni:Co (3:1)	580	115	9.2	142.9	0.6	15	214
**d**	Ni:Co (1:3)	540	131	9.0	138.8	0.63	15.75	222
**e**	Ni:Co (2:1)	490	120	8.9	128.2	0.9	22.5	321
**f**	Ni:Co (1:1)	510	114	8.9	105.4	1.37	34.25	489
**g**	Ni:Co (1:2)	410	112	9.1	98.31	1.42	35.5	507

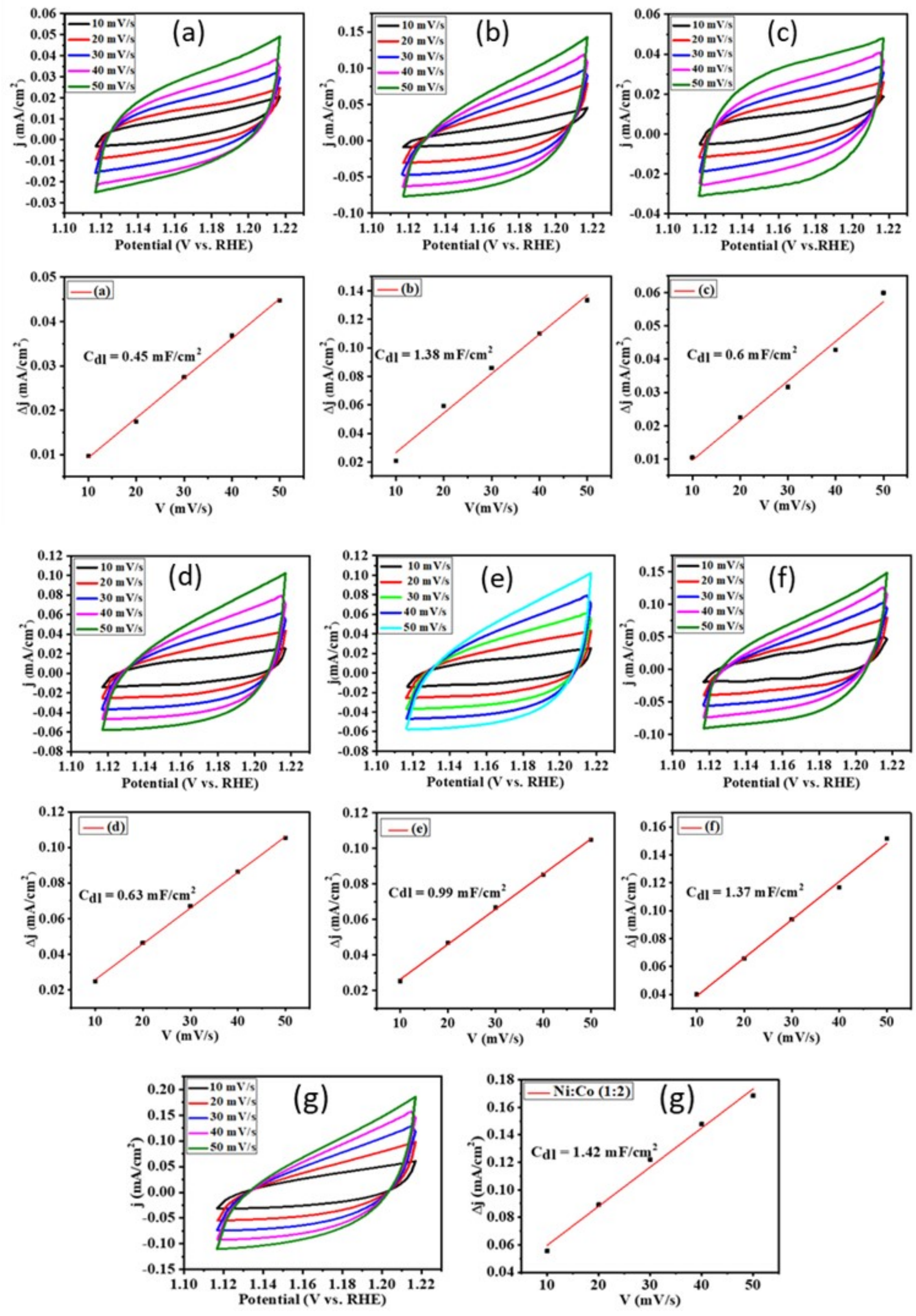

Figure S8: Cyclic voltammograms at scan rate ranging from 10 to 50 mV/s using GCE modified with Ni-Co series (a to g), with corresponding plots (a to g) of current density vs scan rate for ECSA estimation

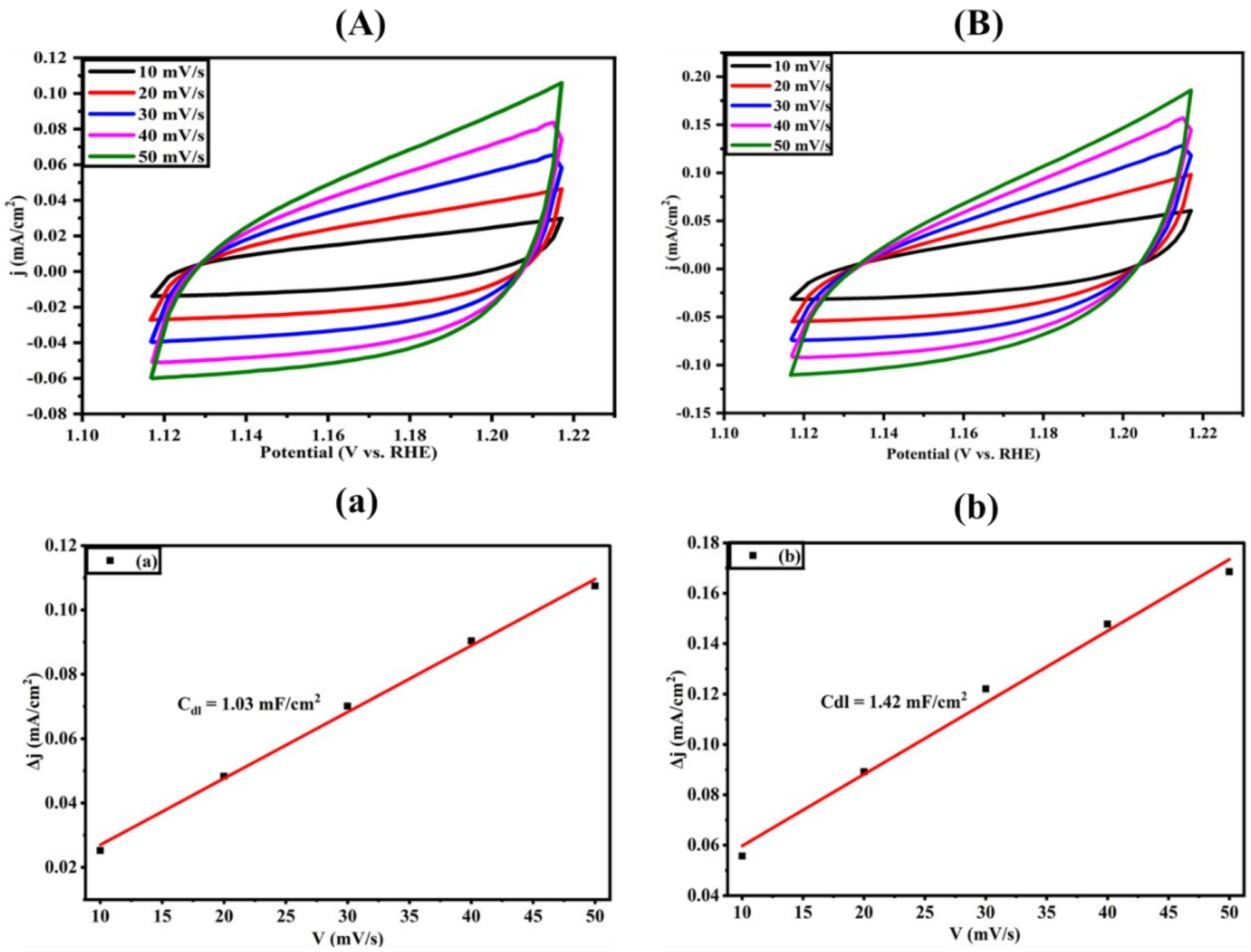

Figure S9: Cyclic voltammogram obtained at GCE modified with **(A)** $FeMoO_4$ (1:1), (**B**) $NiCo_2O_4$ at a scan rate ranging from 10 to 50 mV/s. with corresponding plots of current density vs scan rate **(a)** & **(b)** for ECSA estimation using CV data.

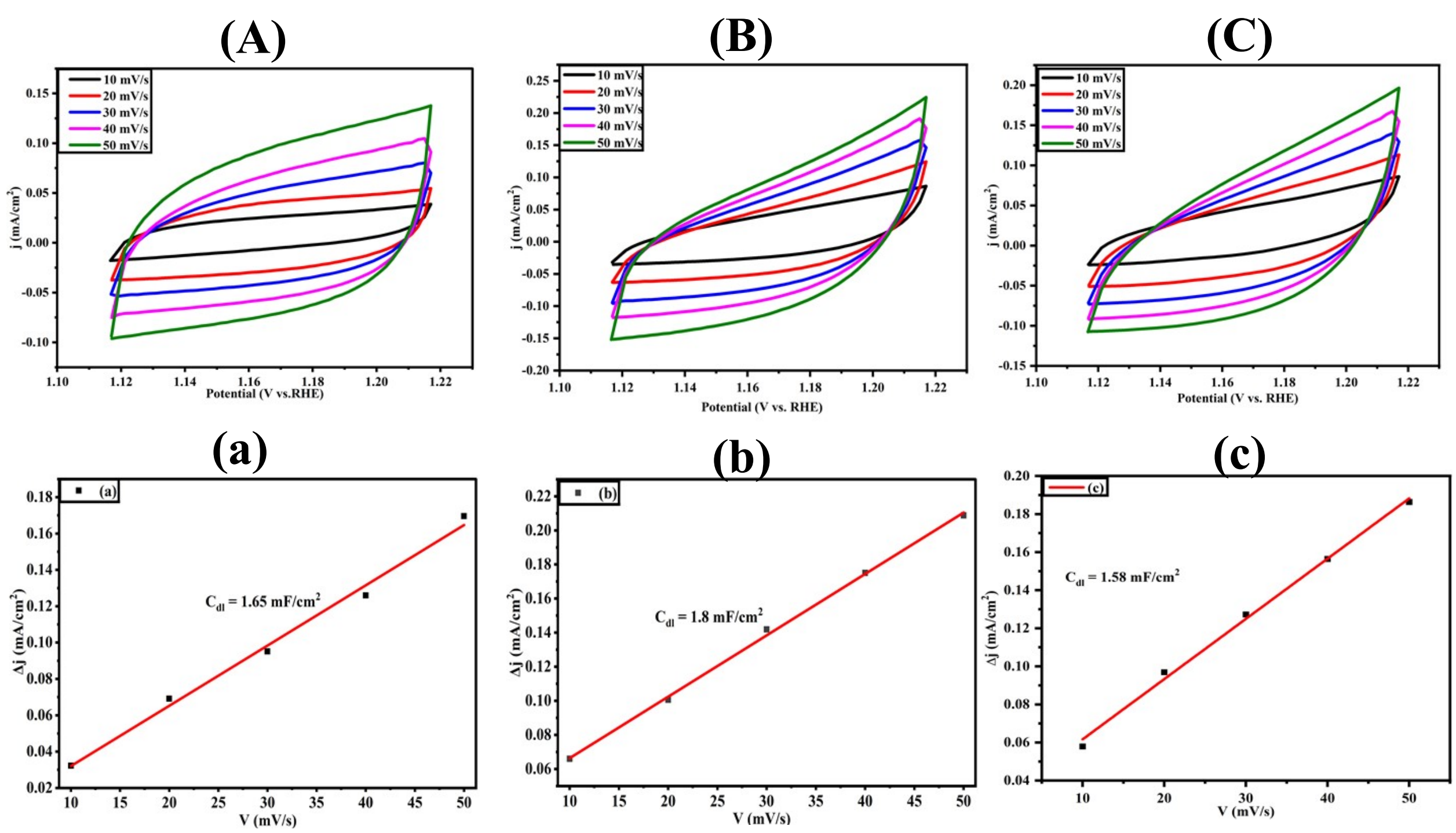

Figure S10: Cyclic voltammogram obtained at GCE modified with **(A)**, **(B)**, and **(C)** $NiCo_2O_4/FeMoO_4$ (3:1, 3:2, 3:3) at a scan rate ranging from 10 to 50 mV/s. with corresponding **(a)**, **(b)**, & **(c)** plots of current density vs scan rate for ECSA estimation using CV data.

Electrochemical Impedance Spectroscopy:

The impedance spectra for both HER and OER were analyzed by fitting to an equivalent-circuit model comprising a solution resistance (R_s) in series with a charge-transfer resistance (R_{ct}) in parallel with a constant phase element (CPE), together with a Warburg diffusion element (W_d) accounting for the mass-transport-limited region of the spectrum. The complete equivalent-circuit diagrams used for the HER and OER fits are given in **Fig. S11** and **Fig. S12**, respectively; the corresponding fitted parameters for all catalysts are collected in **Table S3** (HER) and **Table S4** (OER) below and are discussed in the context of the electrocatalytic HER and OER performance in the main manuscript (**Table 1 and Table 2**).

Table S3. Fitted equivalent-circuit EIS parameters for the HER (0.5 M H_2SO_4).

Electrocatalysts	Solution Resistance	Charge Transfer Resistance	CPE	Wd
Bare Electrode	1.858	5.619	2.05×10^{-4}	2.117
$FeMoO_4$ (1:1)	1.711	5.068	2.36×10^{-4}	1.940
$NiCo_2O_4$ (1:2)	1.759	4.441	3.01×10^{-4}	2.755
$NiCo_2O_4$:$FeMoO_4$ (3:1)	1.797	3.155	3.10×10^{-4}	0.369
$NiCo_2O_4$:$FeMoO_4$ (3:2)	1.878	2.251	2.36×10^{-4}	0.160
$NiCo_2O_4$:$FeMoO_4$ (3:3)	1.809	4.037	3.00×10^{-4}	6.157

Equivalent-Circuit Fitting

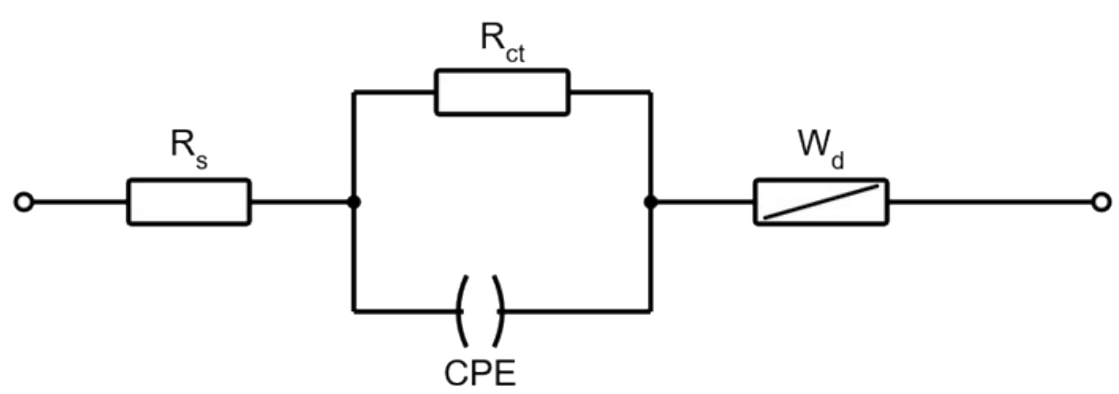

Fig. S11. Equivalent circuit used to fit the HER impedance spectra.

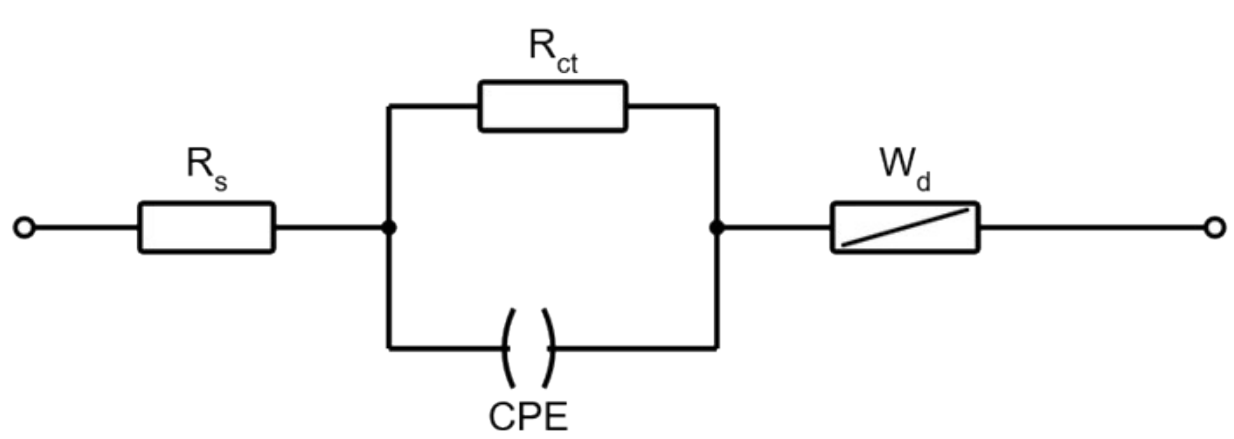

Fig. S12. Equivalent circuit employed for fitting the OER impedance spectra.

Table S4. Fitted equivalent-circuit EIS parameters for the OER (1.0 M KOH).

Electrocatalysts	Solution Resistance	Charge Transfer Resistance	CPE	W_d
Bare Electrode	10.73	166.1	2.35×10^{-6}	46.64×10^{-3}
$FeMoO_4$ (1:1)	9.00	122.8	2.56×10^{-6}	39.05×10^{-3}
$NiCo_2O_4$ (1:2)	8.90	98.31	2.99×10^{-6}	50.56×10^{-3}
$NiCo_2O_4$:$FeMoO_4$ (3:1)	9.98	22.33	14.15×10^{-6}	113.6×10^{-3}
$NiCo_2O_4$:$FeMoO_4$ (3:2)	7.80	19.31	11.42×10^{-6}	132.5×10^{-3}
$NiCo_2O_4$:$FeMoO_4$ (3:3)	6.64	31.54	14.51×10^{-6}	113.6×10^{-3}
